# Purification, Amino Acid Sequence, and Structural Features of a Novel Expansin-like A from the Seeds of Canihua (*Chenopodium pallidicaule* Aellen)

**DOI:** 10.3390/ijms27125213

**Published:** 2026-06-09

**Authors:** Sara Ragucci, Maria Giuseppina Campanile, Rosario Iglesias, Nicola Landi, Claudia Carolina Gay, Angela Oliver, Robina Khan, Lucía Citores, José Miguel Ferreras, Antimo Di Maro

**Affiliations:** 1 Department of Environmental, Biological and Pharmaceutical Sciences and Technologies (DiSTABiF), University of Campania ‘Luigi Vanvitelli’, Via Vivaldi 43, 81100 Caserta, Italy; sara.ragucci@unicampania.it (S.R.); mariagiuseppina.campanile@unicampania.it (M.G.C.); robina.khan@unicampania.it (R.K.); 2 Department of Biochemistry and Molecular Biology and Physiology, Faculty of Sciences, University of Valladolid, 47011 Valladolid, Spain; riglesias@uva.es (R.I.); lucia.citores@uva.es (L.C.); josemiguel.ferreras@uva.es (J.M.F.); 3 Institute of Crystallography, National Research Council, Via Vivaldi 43, 81100 Caserta, Italy; nicola.landi@unicampania.it; 4 Laboratory of Protein Research, Institute of Basic and Applied Chemistry of Northeast Argentina (UNNE-CONICET), Faculty of Exact and Natural Sciences and Surveying, Corrientes 3400, Argentina; claudiacgay@exa.unne.edu.ar; 5 Institute of Biostructures and Bioimaging, National Research Council, Via P. Castellino, 111, 80131 Naples, Italy; angelaoliver@cnr.it

**Keywords:** amino acid sequence, canihua seeds, cell wall loosening, expansin, molecular evolution, protein purification

## Abstract

Expansin-like A (EXLA) proteins belong to one of the four main families within the expansin superfamily, a group of plant proteins essential for cell wall loosening. Here, we report, for the first time, the purification of a novel EXLA, named cpEXLA, from canihua seeds. cpEXLA (yield ~0.16 mg per 100 g of seeds) is a 29 kDa glycoprotein with a high melting temperature (Tm of 86.75 ± 1.06 °C). Elucidation of its primary structure reveals that the mature protein consists of 246 amino acids, ten of which are cysteine residues forming five disulphide bridges. Structural studies based on 3D model prediction reveal the presence of N- and C-terminal domains, which are typical of EXLAs and rich in β-sheets, as confirmed by circular dichroism (CD) spectroscopy. Furthermore, comparative analysis of amino acid sequences between cpEXLA and 219 similar EXLAs, retrieved from dicotyledonous genomes and transcriptomes, identified eighteen invariant amino acid residues: eleven in the N-terminal domain and seven in the C-terminal domain. Finally, phylogenetic analysis of EXLAs in dicotyledonous species shows a close relationship with other EXLAs from the Amaranthaceae family, confirming that EXLA proteins are highly conserved among dicotyledonous plants. Overall, cpEXLA represents an intriguing native tool for studying cell wall evolution and the functional role of EXLAs.

## 1. Introduction

Plants employ various physical and chemical mechanisms for their development [1] and to defend themselves against environmental stress [2], herbivores, and pathogens [2,3], in order to ensure their survival. These mechanisms include the synthesis of new compounds such as alkaloids, glycosides, terpenoids, and proteins/enzymes, as well as the rearrangement of intracellular organelles and the cell wall [4,5,6,7]. Indeed, these mechanisms depend not only on cell biochemical signal networks, but also on cells’ environmental factors due to biological and biophysical constraints. In this scenario, the cell wall plays a central role in the plant life cycle [8]. Indeed, recent studies highlight that the cell wall preserves cellular integrity by resisting the internal hydrostatic pressure [9]. Moreover, this structure has an active role during cell division and differentiation and is a barrier against environmental stress and pathogens [10,11].

A cell wall consists of three main layers: (i) the outer middle lamella, a cementing layer rich in pectin that forms the boundary between adjacent cells; (ii) the primary cell wall, situated inside the middle lamella, which is a flexible layer of cellulose, hemicellulose, and pectin that provides cell protection and growth; and sometimes (iii) the secondary cell wall, a rigid layer deposited between the primary cell wall and plasma membrane, often containing lignin, which provides additional support [12]. Thus, 90–95% of the cell wall total mass is made of cellulose microfibrils, two groups of branched polysaccharides (pectins and cross-linking glycans) and, when present, lignin (a complex phenolic polymer). In addition, different biomolecules (e.g., enzymes and proteins) and ions constituting about 5–10% of the cell wall total mass are involved in the growing and remodeling processes of this rigid layer [13]. In particular, the cell wall proteins (CWPs) are: (i) enzymes (e.g., cellulose synthases, cellulases, arabinosidases, peroxidases, pectinases, and hemicellulases) involved in the reactions that form, remodel, or break down the cell wall structural networks; and (ii) proteins that take part in mechanical strength and expansion, as well as in interactions with the plasma membrane [14,15,16]. Among CWPs, expansins play an important role in the cell wall [17], participating in pH-dependent cell wall remodeling during plant growth and in response to abiotic/biotic stress [18].

Expansins are a family of monomeric proteins of ~25/33 kDa (225–300 amino acid residues), which are ubiquitous in land plants and their algal ancestors, having two structurally conserved functional domains: an N-terminal domain (120–135 amino acid residues) similar to the family-45 glycosyl hydrolase (GH45) and a C-terminal domain (90–120 amino acid residues) similar to the family-63 carbohydrate-binding module (CBM63) [17,19]. The N-terminal region shows a double-psi β-barrel fold domain, while the C-terminal region has a β-sandwich fold. Phylogenetic relationships highlight that the expansin family can be divided into four subfamilies: expansin A (EXPA), expansin B (EXPB), expansin-like A (EXLA), and expansin-like B (EXLB) [13,17,19]. Expansin-like X (EXLX) proteins are found in other organisms like fungi and bacteria [20,21]. The functional role of expansins involves pH-dependent plant cell wall loosening by hydrogen bond breaking between hemicellulose and cellulose microfibrils, without catalytic action [17,18]. In light of this, internal (e.g., phytohormones and osmolytes) and external factors (e.g., developmental stages, pathogens, and environmental stress) regulate the expression pattern of a certain expansin according to its specific function, as reported for *Arabidopsis thaliana* L. [18,22,23].

To date, substantial evidence has linked EXPA and EXPB to cell enlargement, while EXLA and EXLB have received comparatively limited attention [24,25]. Recently, Zhang et al. (2025) cloned four expansin genes from different subfamilies in *Populus tomentosa* TC1521 and characterized their sequences [26]. Heterologous expression in tobacco plants revealed distinct functional roles: *PtoEXPA8* increased leaf area; *PtoEXPB3* promoted floral organ development, earlier flowering, and larger flowers; and *PtoEXLA2* and *PtoEXLB1* increased plant height while reducing flower diameter, with PtoEXLA2 also decreasing 1000-seed weight. Expansins and expansin-like proteins have significant potential in biotechnology because of their ability to modify cell wall properties without polymer degradation. Their expression may contribute to the development of crops with enhanced growth performance, improved tolerance to environmental stresses such as drought and salinity, and increased biomass yield for bioenergy production. Furthermore, the ability of expansins to alter cell wall properties may have implications for improving biomass digestibility, reducing recalcitrance in lignocellulosic feedstocks, and optimizing industrial processes that rely on plant-derived materials [27,28].

In this framework, in an attempt to identify a novel promising biotechnological proteins/enzyme, our group purified and characterized a new expansin, hereafter cpEXLA, from the seeds of *Chenopodium pallidicaule* Aellen. *C. pallidicaule*, known as canihua, is an herbaceous and annual plant native to the Andean region and belonging to the *Chenopodium* genus (Amaranthaceae family) [29], like quinoa [30]. The seeds of canihua are pseudocereal nutritious grains similar to quinoa, considering the crude protein content (with an optimal balance of essential amino acids), fibers, minerals, and a good fatty acid profile [29,31], as well as a wide variety of other health-promoting compounds such as antioxidants, phenols, and flavonoids [32]. The attractiveness of this emerging Andean pseudocereal is also highlighted by the recent studies aimed at the completion of the genome assembly [33]. According to our literature review, cpEXLA is the first purified non-recombinant expansin-like A protein, whose biological activity is still unknown.

Here, we reported the following: (i) physico-chemical properties (molecular weight, far-UV circular dichroism (CD) and thermal stability); (ii) post-translational modifications such as glycosylation; (iii) elucidation of amino acid sequence coupling a strategy based on Edman degradation and cDNA determination by PCR; and (iv) prediction of structure and phylogenetic analysis.

## 2. Results and Discussion

### 2.1. Purification of Novel Expansin-like A

**Figure 1:**
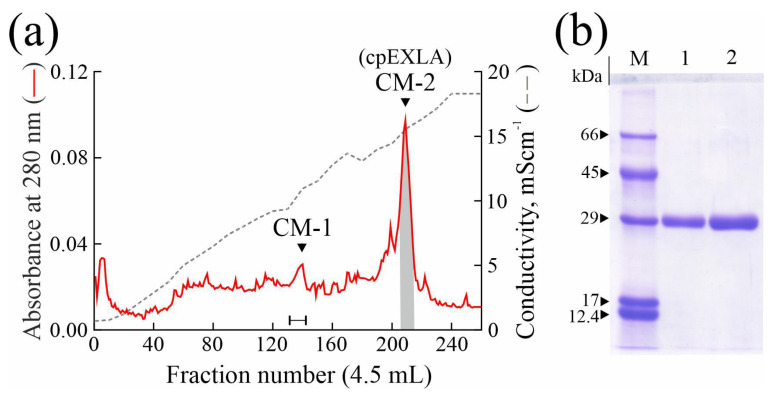
Purification of cpEXLA from *C. pallidicaule* seeds. (**a**) Elution profile of cpEXLA from cation exchange chromatography using CM-Sepharose resin. The shadow area indicates the pooled fractions, corresponding to cpEXLA. (**b**) SDS-PAGE in a 12% polyacrylamide gel of cpEXLA under reducing conditions. Lane M, molecular markers; lanes 1–2, 2.5 µg and 5.0 µg of cpEXLA, respectively.

In order to investigate the presence of novel promising biotechnological proteins/enzymes in *C. pallidicaule*, a standardized procedure for the purification of quinoin, a type 1 ribosome-inactivating protein found in *C. quinoa* seeds [34], which inhibits protein synthesis and exhibits antiviral, antifungal, and anticancer properties [35,36], was undertaken. In this framework, although the crude extracts obtained from the seeds of *C. pallidicaule* slightly inhibited protein synthesis by a rabbit reticulocyte lysate system, we decided to continue the purification procedure, considering the possibility of enriching this inhibitory activity. By the procedure reported in the Materials and Methods, the low inhibitory activity was fractionated by gel-filtration into a single protein peak with an elution volume corresponding to ~29 kDa. The eluted fractions of this peak, containing non-homogeneous proteins, were pooled and subjected to a further step by CM-Sepharose cationic exchange chromatography, using increasing NaCl concentration for the elution. From this last purification step, the pool of 29 kDa was fractionated into two protein peaks: (i) a very broadened peak, barely distinguishable from baseline, named peak CM-1 (fractions 136–144); and (ii) a sharp peak (fractions 207–215), eluted at higher ionic strength, named peak CM-2 (Figure 1a).

In this framework, considering the low content of homogeneous proteins in peak CM-1 fractions analyzed by SDS-PAGE (Figure S1a), separated with low resolution, we decided to characterize only peak CM-2. Thus, each fraction of peak CM-2 was analyzed by SDS-PAGE (Figure S1b), which revealed the presence of a principal protein band (29 kDa) as well as a smear with a molecular weight of ~20 kDa due to a mixture of heterogeneous organic compounds. Nevertheless, extensive dialysis (six changes) of the concentrated pooled fractions against water allowed us to obtain a homogeneous 29 kDa protein. SDS-PAGE analysis of this pool concentrated after dialysis highlights the presence of a single protein band with an apparent relative molecular mass (M*r*) of ~29 k (Figure 1b). The homogeneity of this purified protein was also confirmed by analytical RP-HPLC (Figure S1c).

Finally, this pool, hereafter cpEXLA, was aliquoted and stored at −80 °C until further experiments. The average yield from five different purification procedures was ~0.16 mg of cpEXLA per 100 g of *C. pallidicaule* seeds.

### 2.2. Effect of cpEXLA on Protein Synthesis

The effect of purified cpEXLA on protein synthesis using a rabbit reticulocyte lysate system was evaluated. However, this protein exhibited a low capability to inhibit protein synthesis in vitro, showing an IC_50_ value (concentration causing 50% inhibition) of 5 µM. On the other hand, cpEXLA does not exhibit either ribonuclease activity on high-molecular-weight yeast RNA (Figure S2a) or polynucleotide:adenosine glycosylase (PNAG) activity, differently from quinoin [34,37] when salmon sperm DNA is used as a substrate (Figure S2b).

### 2.3. Structural Features and N-Terminal Amino Acid Sequence of cpEXLA

**Figure 2:**
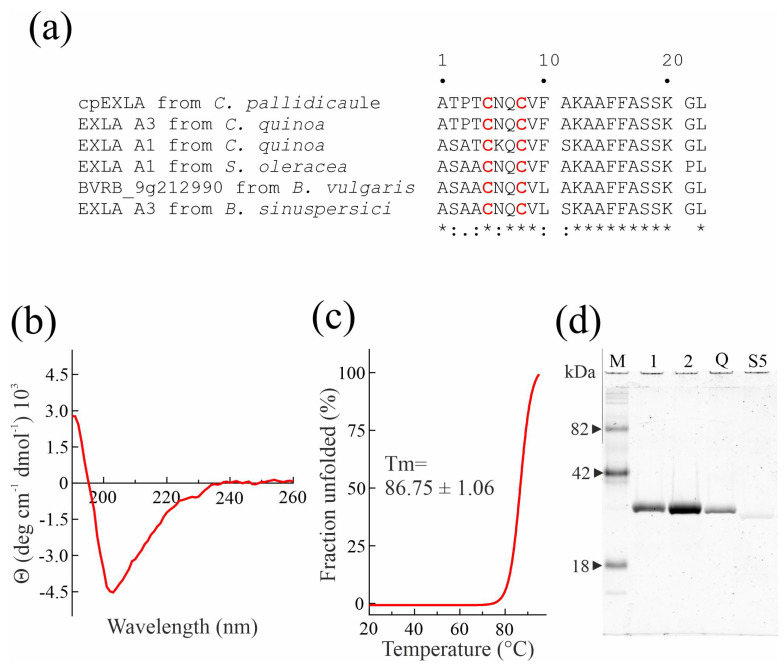
Structural features of cpEXLA from *C. pallidicaule* seeds. (**a**) N-terminal amino acid sequence alignment among cpEXLA and expansin-like proteins isolated from plants. Expansin-like A3 from *Chenopodium quinoa* (XP_021774691); expansin-like A1 from *Chenopodium quinoa* (XP_021773664); expansin-like A1 from *Spinacia oleracea* (XP_056699025); hypothetical protein BVRB_9g212990 from *Beta vulgaris* subsp. *Vulgaris* (KMT01282), and expansin-like A3 from *Bienertia sinuspersici* (KAL2936922). ‘‘*’’ identical, ‘‘:’’ conserved, and ‘‘.’’ semi-conserved amino acid residues. In red, cysteinyl residues. (**b**) Far-UV CD spectrum of cpEXLA (0.15 mg mL^−1^). (**c**) Thermal denaturation profile of cpEXLA (0.07 mg mL^−1^). The fraction unfolded at 278 nm is plotted as a function of temperature. (**d**) In gel staining for sugars of cpEXLA. Lane M, CandyCane ™ glycoproteins molecular weight standards; lane Q, 3.0 μg of quinoin (N-glycosylated type-1 RIP from *Chenopodium quinoa* seeds); lane S5, 3.0 μg of sodin 5 (non-glycosylated type-1 RIP from *Salsola soda* seeds); lanes 1 and 2, 2.5 and 5.0 μg of cpEXLA, respectively.

In order to quickly obtain functional or biological information about cpEXLA, considering the absence of the tested enzymatic activities, we decided to determine the N-terminal sequence of this protein. Edman degradation analysis on S-pyridylethylated cpEXLA allowed us to determine the first 22 amino acid residues at the N-terminal, as reported in Figure 2a. This query was submitted to the *C. pallidicaule* assembled genome database for sequence homology studies. The search revealed the highest percentages of identity/similarity with the N-terminal amino acid sequence of expansins, which are proteins involved in the loosening of plant cell walls by hydrogen bond breaking between hemicellulose and cellulose microfibrils [17,18].

In light of this, to confirm structurally that cpEXLA belongs to the expansin family, we performed a far UV circular dichroism (CD) analysis in order to obtain information on the secondary structure of cpEXLA. The acquired spectrum is reported in Figure 2b and shows the presence of a positive peak at ~195 nm and a negative peak at ~205 nm, suggesting that the secondary structure of cpEXLA is dominated by β-strands. Thus, this finding highlights that cpEXLA shows a similar fold to that of most expansins [38]. Subsequently, the thermal denaturation curve of cpEXLA was obtained using UV-spectroscopy by measuring the increment in absorbance at 278 nm as the temperature was increased. The melting temperature (Tm) of cpEXLA was 86.75 ± 1.06 °C (Figure 2c), highlighting that this biomolecule is a highly stable protein. In addition, a specific glycoprotein detection analysis revealed that cpEXLA is a glycoprotein when analyzed by SDS-PAGE and sugar staining under reducing conditions (Figure 2d). The glycosylation of cpEXLA was also confirmed by mass spectrometry analysis. Indeed, cpEXLA desalted by RP-HLPC exhibits a heterogeneous mass spectrum since the intact mass spectrum revealed the presence of several cpEXLA glycoforms (Figure S3).

Furthermore, to evaluate the number of cpEXLA cysteinyl residues, considering the high number of disulfide bonds present in the expansin family [38], we determined the amino acid composition of this protein with and without performic acid pre-treatment followed by hydrolysis with HCl. The experimental residues per mole of protein are reported in Table S1. In particular, cpEXLA has ~10 cysteines per mole of protein. Moreover, the amino acid analysis shows that cpEXLA has ~34.8% of aliphatic amino acids, ~8.4% of glycine, ~6.4% of proline, and ~10.8% of basic amino acids (lysine + arginine). On the other hand, the negative reactivity of cpEXLA with Ellman’s reagent suggests that the cysteinyl residues of this protein are involved in forming disulfide bridges [39].

### 2.4. Primary Structure of cpEXLA

To gain insight into the identity and structure of cpEXLA, its N-terminal sequence was then used as a query to search for similar sequences using BLAST (version BLAST+ 2.17.0) in the NCBI database. The sequence matched sequence MATR01000013 (*C. pallidicaule* isolate PI_478407 scaffold_38, whole genome shotgun sequence). Based on this sequence and the amino-terminal sequence determined by Edman degradation, specific primers were designed for the amplification of cpEXLA cDNA (see Section 3.12).

PCR fragments of approximately 1800 and 800 bp were generated (Figure S4) and cloned into the pCR^TM^ II vector. The sequences of five overlapping fragments were determined. Three of them showed the presence of four introns, with consensus splice sequences and stop codons within the intron, and two of them lacked introns and had an open reading frame. The presence of introns could be attributed to genomic DNA contamination or the presence of unprocessed mRNA. Furthermore, intron retention is a frequent phenomenon linked to regulatory mechanisms [40]. Studies have shown that intron distribution patterns in EXPA, EXPB, and EXLA/EXLB are highly conserved among land plants, angiosperms, and seed plants, respectively [20]. In EXLA and EXLB, these patterns are generally conserved, characterized by the presence of three introns in the DPBB domain and one intron in the CBM63 domain (Pattern V), except for EXLBs in eudicots, which exhibit Pattern IV, containing two and one introns in the DPBB and CBM63 domains, respectively.

**Figure 3:**
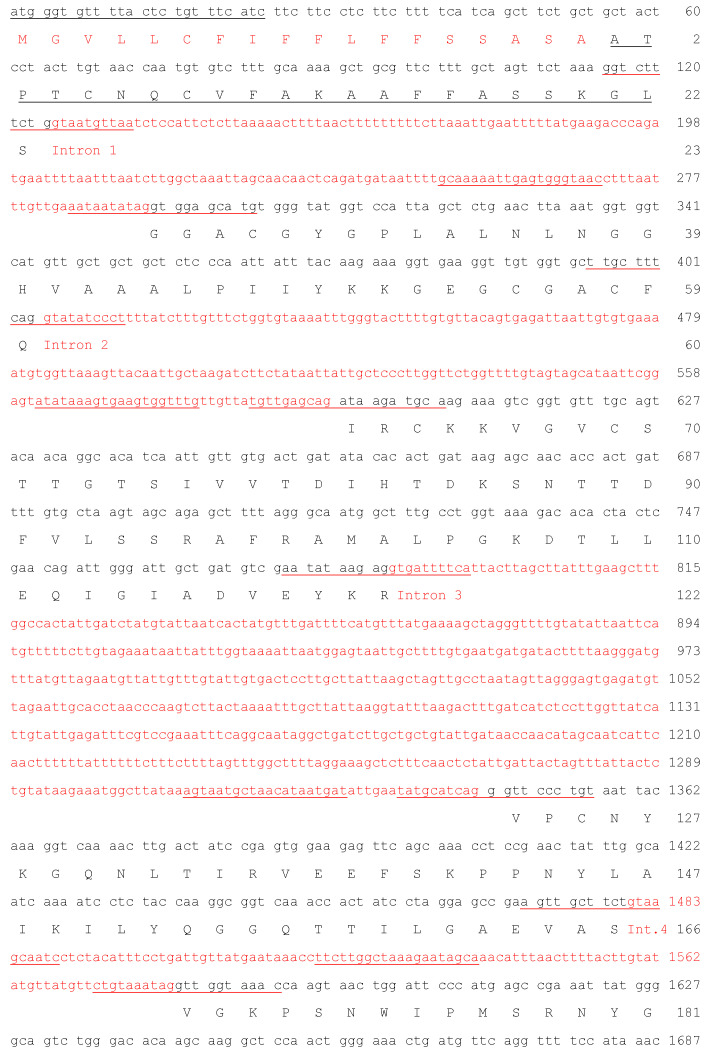
Complete nucleotide and amino acid sequences of cpEXLA. The cDNA sequence of cpEXLA was determined as described in Section 3.12. Primers and the amino-terminal sequence identified by Edman degradation are underlined, and introns are shown in red. The consensus sequences of the donor and acceptor splice sites, as well as branch points, are underlined in red. The numbering of nucleotides (of the complete gene) and amino acids (of the mature protein) is shown on the right. The DNA sequence of cpEXLA was submitted to GenBank (accession number: PZ166062).

The sequenced cDNA consists of 1822 bp and contains 4 introns, which, once removed, produced a 792 nt RNA encoding a 264 amino acid pre-protein in which 18 amino acids were removed from the amino terminus to produce the mature 246 amino acid protein (Figure 3), with a theoretical molecular weight (MW) of 26,530.74 Da, which matches the molecular mass determined by SDS-PAGE.

In light of this, it is possible to justify the presence of A-H isoforms in the deconvoluted cpEXLA mass spectrum (Figure S3). The theoretical MW of mature cpEXLA (26,530.74 Da) is calculated based on 10 reduced cysteinyl residues. However, since Ellman’s assay did not detect free sulfhydryl groups, the MW is 26,520.74 Da, considering −2 Da for each disulfide bridge (−10 Da). Considering the above, the GlycoMod tool [41] predicted that the N-glycan chain of glycoform A (27,569.07 Da) is [(Hex)_3_ (HexNAc)_2_ (Pent)_1_] (1024.93 Da), as was found in other proteins extracted from plant seeds [42,43,44]; while the other glycoforms (B–H) can be explained by the addition of N-Acetyl-hexosamine, hexoses, and pentoses with or without methionine sulfoxide or methionine sulfone, as shown in Table S2.

### 2.5. Prediction of the Structure of cpEXLA

**Figure 4:**
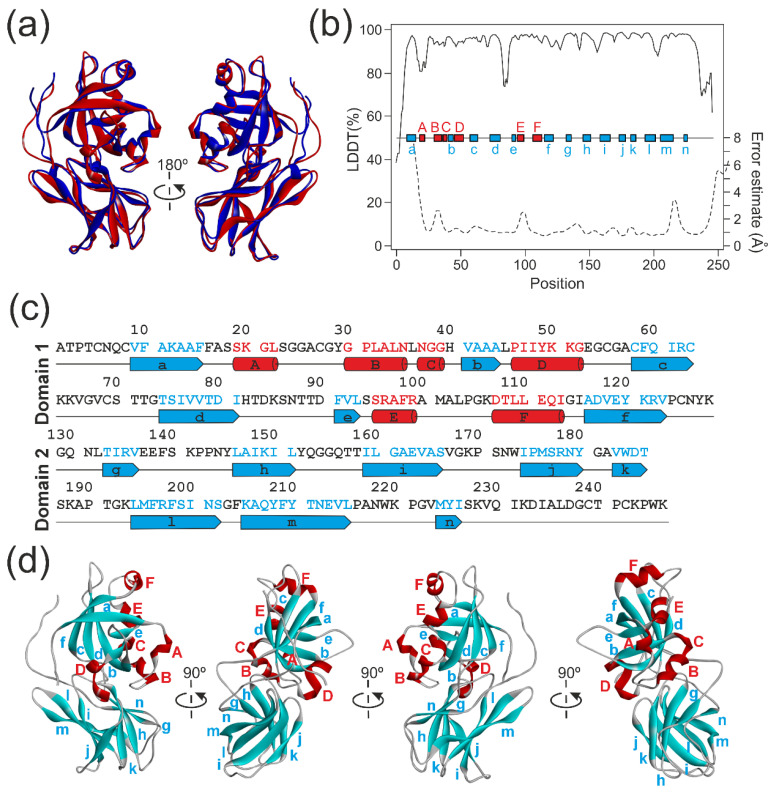
Prediction of the three-dimensional structure of cpEXLA. (**a**) Comparison of the predictions of the structure of cpEXLA made by AlphaFold2 (red) and RoseTTAFold (blue). (**b**) Confidence of the models predicted by AlphaFold2 and RoseTTAFold. The local distance difference test (LDDT) performed by AlphaFold2 (solid line) and the estimated error performed by RoseTTAFold (dashed line) are shown for each position in the protein polypeptide chain. The boxes indicate the secondary structure (cyan ribbons, β chains; red cylinders, helices) of each position predicted by AlphaFold2. (**c**) The secondary structures of cpEXLA are shown in red (helices, labeled A to F) and cyan (β chains, labeled a to n). (**d**) Three-dimensional structure of cpEXLA predicted by AlphaFold2. Helices (red) and β chains (cyan) are shown. The helices are labeled A to F, and the β chains are a to n.

The structure of cpEXLA was predicted using the AlphaFold2 [45] and RoseTTAFold [46] programs. As shown in Figure 4a and Figure S5, despite using different algorithms, the two programs predicted very similar structures.

The biggest differences between the two predictions occurred in the loops of the amino-terminal region (amino acids 1 to 8) and carboxyl-terminal region (amino acids 233 to 246), while the two predictions agree on the structures of the helices and β-strands (Figure 4a). Both models showed excellent confidence scores, with LDDT values above 90% for the AlphaFold model and RMSD values below 2 Å for the RoseTTAFold model (Figure 4b). This allows these models to be used for molecular-level studies [45,46]. The exceptions were the aforementioned amino and carboxyl ends, helix A, and the loops between chains d and e (Figure 4b).

cpEXLA has a globular structure with two clearly differentiated domains (Figure 4c,d), as has already been described for other expansins [47]. In fact, it has 60% identity, an RSDM of 1.03, and a TM-score of 0.83 with GhEXLA1 (Figure S6), an EXLA obtained from *Gossypium hirsutum*, which, although not published, is in the Protein Data Bank with accession number 7XC8. Among published proteins, the most similar is EXPB1 (Zea m 1) obtained from corn [48], with which it shares 32% identity, an RSDM of 2.21, and a TM-score of 0.83 (Figure S6).

The N-terminal domain is a six-chain double psi beta barrel (DPBB) [49], containing approximately 128 amino acid residues (Figure 4c,d). This domain shares some similarities with the catalytic domain of the glycosidase hydrolase 45 (GH45) family protein, and the DPBB is sometimes also referred to as GH45-like [8]. However, the β-1,4-glucanase activity of the GH45 enzyme has not been detected in expansins, and the GH45 enzyme also lacks the wall-extending activity of expansin [48].

The carboxyl-terminal domain includes a CBM63 fold (amino acids 129–227) followed by a 19-amino acid loop. The CBM63 fold consists of two sets of four antiparallel β chains forming a β sandwich [50]. The CBM63 fold is present in all expansins, while the carboxyl-terminal extension is unique to EXLA [38].

### 2.6. Homology of cpEXLA with Other Hypothetical Proteins

**Figure 5:**
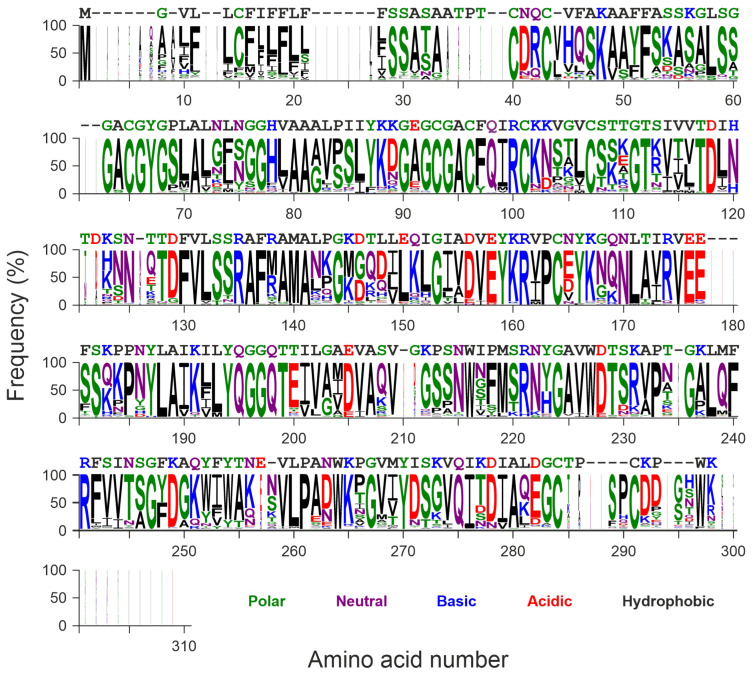
EXLAs sequence logo. The sequence logo representation of the alignment of 220 EXLA precursor sequences was created as described in Section 3.14. The height of the letters is proportional to the frequency of each amino acid at that position in the alignment with respect to all amino acids; the width of the letters is proportional to the frequency of an amino acid, including gaps. The sequence of the cpEXLA precursor is shown above the logo. The colors indicate the classes of amino acids according to their chemical properties: hydrophobic (black), polar (green), neutral (purple), acidic (red), and basic (blue).

Using the sequence of the precursor of cpEXLA, a database search yielded 219 results, all of which were sequences of unknown proteins obtained by conceptual translation of dicotyledonous genomes or transcriptomes, while no monocotyledonous sequences were obtained. Figure 5 displays a logo produced by aligning the 220 sequences shown in the Supplementary Material (Figure S7).

Eleven amino acids in domain 1 remain unchanged (C40, C43, G63, C65, C93, C96, C101, C107, G111, A141, and C164, in the logo), while there are seven invariant amino acids in domain 2 (Q195, G196, G236, G247, P261, Y270, and C291, in the logo). In addition, many amino acids are close to 100% frequency, especially those in domain 1. Therefore, it appears that EXLAs have unusually homogeneous sequences throughout the dicotyledonous class.

The expansin gene family is classified into four major subfamilies: EXPA (first described), EXPB, EXLA, and EXLB proteins. Members of the EXPA group are characterized by a conserved His-Phe-Asp (HFD) motif located at the N-terminus of domain I. In contrast, EXLAs contain a Thr-Asp-Phe (TDF; also present in the EXLB subfamily) and Cys-Asp-Arg-Cys (CDRC) motifs and display an extended domain II (cpEXLA has 20 additional amino acids) [38]. However, in cpEXLA, the CDRC motif described by these authors in domain I is replaced by Cys-Asn-Gln-Cys (CNQC), suggesting that the Cys residues conserved at positions 40 and 43 are characteristic of this subfamily, rather than the amino acids at intermediate positions, which may be charged or neutral (Figure 5). The DPBB domain also contains the GACG and CGAC motifs, which are separated by 26 amino acids and are highly conserved in the four subfamilies of expansins in plants [20].

### 2.7. Phylogenetic Relationship Among cpEXLA and Other Expansins

**Figure 6:**
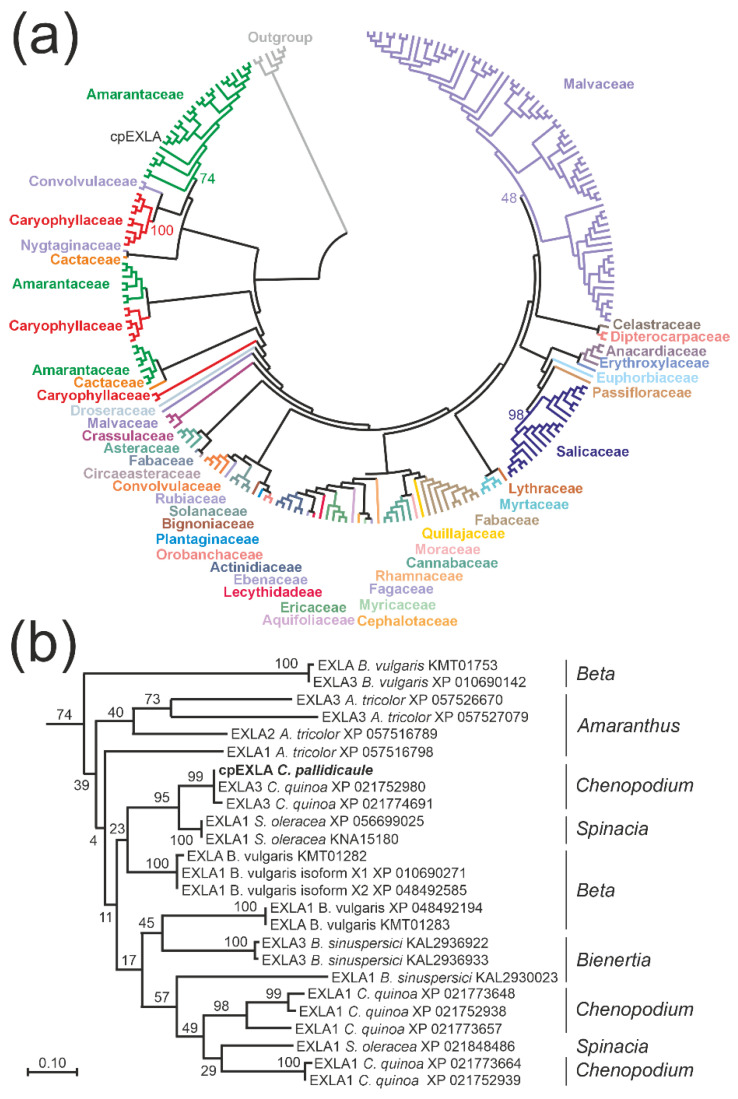
Phylogenetic relationship of cpEXLA with amino acid sequences of hypothetical protein precursors derived from the genomes of various angiosperms. (**a**) Phylogenetic analysis using the maximum likelihood method of the precursors of expansin-like protein A (EXLA) in angiosperms. The evolutionary history was deduced as indicated in Section 3.14. Next to the branches of families with the highest number of sequences, the percentage of trees in which the associated taxa are grouped is shown. The detailed phylogenetic tree is shown in Figure S7. (**b**) Detail of the Amaranthaceae clade in which cpEXLA is found. The sequence name, species, genus, family, and accession number are indicated.

Within the dicotyledonous angiosperms, after removing sequences with long signal peptides, the 220 sequences of EXLAs belonging to 38 families of 18 orders (Table S3) were used for the phylogenetic study shown in Figure 6a, including an outgroup with six EXPA precursors (Figure S8). The closest sequences in the phylogenetic tree are XP_021752980, identical to cpEXLA, and XP_021774691, which differs from it in only four amino acids (98.48% of identity), both belonging to *C. quinoa* (quinoa), which, together with *C. pallidicaule*, are part of the Amaranthaceae family (Figure 6b). Quinoa (like canihua) is a pseudocereal well-adapted to drought and poor soils in marginal environments [51].

Other genera of the family, such as *Beta vulgaris* (beetroot) L. and *Spinacia oleracea* L. (spinach), which belong to the same clade, showed percentages of identity with cpEXLA ranging from 83.67% to 90.38%, respectively. Furthermore, sequences from different clades of *Bienertia sinuspersici* Akhani (a C4 desert plant) showed a degree of identity with cpEXLA ranging from 68.85% to 78.17%.

## 3. Materials and Methods

### 3.1. Chemicals and Reagents

The chemicals used in this work were previously reported [34,52,53,54], and most of them were obtained from Sigma-Aldrich Solutions (Merk Life Science, Milan, Italy). Bovine pancreatic ribonuclease A (RNase A) and yeast RNA were purchased from Roche Diagnostics S.L. (Barcelona, Spain). Century ™-Plus RNA Markers were purchased from Fisher Scientific (Madrid, Spain).

The following buffers in MilliQ water (Millipore Co., Milan, Italy) were used: buffer A: 5.0 mM Na-phosphate, pH 7.2 containing 0.14 M NaCl; buffer B: 10 mM Na-acetate, pH 4.0; buffer C: 5.0 mM Na-phosphate, pH 7.2; and buffer D: 5.0 mM Na-phosphate, pH 7.2, containing 0.3 M NaCl.

### 3.2. Plant Material

*C. pallidicaule* seeds were purchased in a local market of Corrientes (Corrientes, Argentina). Seeds, gently cleaned with a sieve, were stored at −80 °C until use.

### 3.3. Protein Purification

Seeds (200 g) were homogenized in 1 L (*w*:*v*; 1:5) of buffer A in a Waring blender (Waring Products; Torrington, CT, USA). The homogenate was stirred for 12 h at 4 °C, filtered through Miracloth paper (Inalco, Milan, Italy), and centrifuged at 15,000 *g* (Centrifuge Avanti J-25, Beckman Coulter, Brea, CA, USA), at 4 °C for 1 h. The pH of the crude extract was adjusted to pH 4.0 with glacial acetic acid, stirred at 4 °C for 1 h, and then centrifuged at 15,000 *g* (Centrifuge Avanti J-25), at 4 °C for 1 h. The supernatant, further filtered through Miracloth paper, was loaded onto a Streamline ™ SP column [L × I.D. 15 × 5 cm; Cytiva, Buccinasco (MI), Italy], and equilibrated in buffer B at a flow rate of 3.0 mL/min. After sample loading, the column was sequentially washed in buffer B and buffer C until the absorbance at 280 nm was below 0.01 optical density (O.D.). Bound basic proteins were eluted with buffer C containing 1 M NaCl, monitoring the absorbance of the eluate at 280 nm. Fractions (each 10 mL) were pooled and concentrated in an Amicon cell concentrator (200 mL) fitted with a PM-10 membrane (MWCO 10,000 Da). The resulting basic protein sample (15 mL) was subjected to gel-filtration using a Hi-Load^®^ 26/600 Superdex^®^ 75 pg column (L × I.D. 60 cm × 26 mm; Cytiva; 3000–75,000 separation range) by FPLC on AKTA Purifier System (GE Healthcare, Uppsala, Sweden), equilibrated with buffer D. Proteins were eluted with the same buffer at a flow rate of 2.5 mL/min. Fractions (5.0 mL) with elution time corresponding to ~30 kDa were pooled, dialyzed against buffer C (three times), and loaded onto CM-Sepharose (L × I.D. 15 × 1.6 cm; Cytiva) column, equilibrated with buffer C. Proteins were eluted with a 0.0–0.22 M NaCl linear gradient in C (500 mL buffer A 5.0 mM Na-phosphate, pH 7.2; 500 mL buffer B 5.0 mM Na-phosphate containing NaCl 0.22 M, pH 7.2; total volume 1 L; flow rate 0.6 mL/min) by using a peristaltic pump. Finally, in order to eliminate the presence of organic compounds from the protein peak of interest, an extensive dialysis against deionized water was carried out using a membrane Spectra/Por^®^ 1 MWCO 6000/8000 (Repligen Corporation, Waltham, MA, USA).

During the purification procedure, the protein homogeneity was verified by SDS-PAGE under reducing conditions.

### 3.4. Analytical Procedures

Purity and integrity of cpEXLA were determined by SDS-PAGE [55] with a Mini-Protean III mini-gel apparatus (Bio-Rad, Milan, Italy) using a 6% stacking gel and a 12% separating gel. BSA (66 kDa), ovalbumin (45 kDa), carbonic anhydrase (29 kDa), myoglobin (16.9 kDa), and cytochrome C (12.4 kDa) were used as molecular weight markers. Protein concentration was determined by the Pierce BCA Protein Assay kit (Thermo Fisher Scientific, Rodano, Italy), using BSA as standard [56]. Pro-Q^TM^ Emerald 300 Glycoprot Probes Kombo (Life Technologies Italia; Segrate (MI), Italy) was used to determine the glycosylated proteins by in-gel analysis after SDS-PAGE. Glycosylated proteins were visualized using a ChemiDoc^TM^ XRS system (Bio-Rad). The quantification of free sulfhydryl groups in solution by Ellman’s assay was carried out using DTNB [5,5′-dithio-bis-(2-nitrobenzoic acid)] [39].

### 3.5. Reduction and S-Pyridylethylation

S-pyridylethylation (alkylation of cysteinyl residues with 4-vinylpyridine) after reduction with β-mercaptoethanol was performed as previously reported [52]. The alkylated desalted protein was obtained by RP-HPLC using a BioBasic-4 column (150 × 4.6 mm, 5 µm particle size; Thermo Fisher Scientific, Rodano, Italy) at 25 °C. Solvents used were Milli-Q water containing 0.1% TFA (solvent A) and acetonitrile containing 0.1% TFA (solvent B) [57]. Protein elution was performed using a linear gradient of solvent A and solvent B, from 5% to 65% of solvent B over 60 min (flow rate of 1.0 mL/min), monitoring the absorbance at 214 nm.

### 3.6. Effect on Protein Synthesis

The effect on protein synthesis was analyzed as described by Iglesias et al., 2022 [54], through a coupled transcription–translation in vitro assay using a rabbit reticulocyte lysate system. Data represent the average of five independent experiments performed in duplicate.

### 3.7. Enzymatic Assays

#### 3.7.1. Polynucleotide: Adenosine Glycosylase Activity (PNGA) on Salmon Sperm DNA

The adenine release was measured as previously reported [58,59], incubating salmon sperm DNA (100 µg) with proteins (5.0 µg) in 300 µL 50 mM sodium acetate (pH 4.0) containing 100 mM KCl, at 30 °C for 1 h. After incubation, the DNA was precipitated with cold ethanol and centrifuged. Adenine release was determined spectrophotometrically, reading the supernatant at 260 nm.

#### 3.7.2. Ribonuclease Activity on High-Molecular-Weight Yeast RNA

Ribonuclease activity was evaluated as previously reported [60]. Briefly, reaction mixtures of 0.2 mg/mL high-molecular-weight yeast RNA in 0.1 M 3-N-morpholino propanesulfonic acid (MOPS) pH 7.5, containing 2.0 mM EDTA and 10 µg/mL methylene blue, were incubated for 60 min at room temperature in the presence of cpEXLA (10,000 ng/mL) or different concentrations of ribonuclease A (EC 3.1.27.5) from bovine pancreas (800, 80, 8.0, 0.8, and 0.08 ng/mL), used as a reference protein. The shift in the maximum absorbance of methylene blue upon intercalation into high-molecular-weight yeast RNA was followed spectrophotometrically at a wavelength of 688 nm after 0.0, 15, 30, 45, and 60 min.

### 3.8. Automatic N-Terminal Edman Degradation and Sequence Comparison

The N-terminal sequence of S-pyridylated protein was obtained by using automated Edman degradation carried out on a Shimadzu PPSQ 33B sequencer (Shimadzu Italia S.r.l., Milan, Italy) through the service of ‘Protein/Peptide sequencing’ from the Institute of Biosciences and Bioresources (IBBR-CNR, Naples, Italy). The protein was first alkylated with 4-vinylpyridine, desalted by RP-HPLC (as described in Section 2.5), freeze-dried, and loaded on the sequencer.

The N-terminal amino acid sequences similar to that of cpEXLA and used in this study were retrieved using the BLASTp tool at NCBI (https://blast.ncbi.nlm.nih.gov/Blast.cgi?PAGE=Proteins (accessed on 10 February 2025)) and downloaded from GenBank (https://www.ncbi.nlm.nih.gov/genbank/ (accessed on 10 February 2025)). Multiple alignment was performed by ClustalW at EMBL-EBI (https://www.ebi.ac.uk/jdispatcher/msa/clustalo (accessed on 10 February 2025)).

### 3.9. Amino Acid Analysis

Amino acid analyses were performed by using a Biochrom 30 amino acid analyzer (Biochrom, Cambridge, UK), using *nor*-Leu as an internal standard [58,61]. In addition, to detect the cysteine content, protein was subjected to oxidation with performic acid as previously reported [62]. All experiments were performed in triplicate.

### 3.10. Circular Dichroism and Thermal Stability Determination

The far-UV CD spectrum of cpEXLA was acquired at 25 °C on a Jasco J-815 dichrograph [Jasco Europe, Cremella (LC), Italy]. A protein concentration of 0.15 mg/mL (~5.0 µM) in 10 mM Na-phosphate, pH 7.2 (path-length quartz cuvette of 0.1 cm), was used for the far-UV spectrum measurements [63]. DichroWeb (online analysis for protein circular dichroism spectra; https://bio.tools/dichroweb (accessed on 15 October 2025) [52] was used to estimate the percentages of secondary structural elements.

The thermal stability of cpEXLA was determined by monitoring the signals at 278 nm on a UV-VIS Cary 100 spectrometer [Agilent Technologies Italia S.p.A., Cernusco sul Naviglio (MI), Italy], equipped with a Peltier temperature controller. The protein (~0.07 mg/mL; ~2.5 µM) in 10 mM Na-phosphate, pH 7.2, was subjected to heat-induced denaturation, raising the temperature from 20 °C to 95 °C at a rate of 1 °C/min. The fraction that unfolded was calculated from observed absorbance and plotted against temperature. The midpoint temperature of the unfolding curve was determined by data fitting to the Boltzmann model using Prism 8 (GraphPad Software Inc., San Diego, CA, USA).

### 3.11. Electrospray Mass Spectrometry (ESI-MS) Analysis

The protein was analyzed by LC-MS using a ThermoFisher system (Thermo Fisher Scientific, Germering, Germany) equipped with a quaternary pump, an automated autosampler, a multiwavelength Ultimate 3000 Diode Array detector, and a LTQ Linear Ion Trap mass spectrometer, as previously reported [64]. Multicharged spectra were deconvoluted using BioPharma Finder, ver. 5.1 (ThermoFisher Scientific, Rodano, Italy), using the ReSpect algorithm for isotopically unresolved intact protein analysis.

### 3.12. Synthesis, Cloning, and Sequencing of cDNA

Canihua germ (100 mg) was crushed in liquid nitrogen until a fine powder was obtained, and total RNA was isolated using the NucleoSpin^®^ RNA kit (Macherey-Nagel, Düren, Germany). This procedure included treatment with DNase to completely remove any traces of genomic DNA. Poly(A)-rich RNA was reverse transcribed using synthetic oligonucleotide T1 (5′ CGTCTAGAGTCGAGTCGACTAGTGC(T)_20_ 3′), following a procedure described previously [65]. Specific primers were designed for the cpEXLA gene sequence based on the N-terminal sequence obtained by Edman degradation and the MATR01000013 sequence (*C. pallidicaule* isolate PI_478407 scaffold_38, whole genome shotgun sequence): cpEXLAf (5′ ATGGGTGTTTTACTCTGTTTCATC 3′) as the forward primer and cpEXLAr (5′ TTTCCATGGTTTACAAGGAGTGC 3′) as the reverse primer. cDNA amplification was performed as described previously [65]. PCR amplification was performed under the following conditions: initial denaturation at 94 °C for 3 min, followed by 35 cycles of 94 °C for 30 s, 54 °C for 30 s, and 72 °C for 60 s, and an additional extension of 10 min at 72 °C. PCR products of approximately 1800 and 800 bp (Figure S4) were produced, purified, ligated into the pCR^TM^ II vector (Invitrogen, Thermo Fisher Scientific, Alcobendas (Madrid), Spain), and subsequently used to transform chemically competent *E. coli* DH5α bacteria (Invitrogen). Five clones, three of which were long (1800 bp) and two of which were short (800 bp), were purified and sequenced. Thus, the complete sequence of the gene encoding cpEXLA with and without introns was determined by overlapping sequences to produce intron-free cpEXLA coding cDNA. The cpEXLA cDNA sequence was submitted to GenBank (accession number: PZ166062).

### 3.13. Prediction of the Structure of cpEXLA

The structure of cpEXLA was predicted using the AlphaFold2 [45] and RoseTTAFold [46] structure prediction tools. Of the five models proposed by each program, those with the most accurate prediction parameters were selected. The alignment and comparison of protein structures was performed using the TM-align algorithm [66] on the website https://www.rcsb.org/alignment (accessed on 19 October 2025). Secondary structures were assigned using DSSP [67,68] at https://pdb-redo.eu/dssp (accessed on 22 November 2025). Representations and graphics from the protein structure study were generated using the Discovery Studio Visualizer (v21.1.0) package (https://www.3dsbiovia.com/) (accessed on 26 April 2022).

### 3.14. Sequence Alignment and Phylogenetic Analysis

The amino acid sequences were obtained from the National Center for Biotechnology Information (https://www.ncbi.nlm.nih.gov/protein/) (accessed on 20 February 2025). Sequence alignment and graphical representation of the logo were performed using MEGA 11 [69] and WebLogo 3 [70] software, respectively. Phylogenetic analysis was performed using MEGA 11 software [69] with the maximum likelihood method [71]. Plant species were named and classified according to the Angiosperm Phylogeny Group (APG) classification [72], as consulted in the WFO Plant List (https://wfoplantlist.org/) (accessed on 5 November 2025).

## 4. Conclusions

This study established, for the first time, a robust biochemical procedure to purify cpEXLA, a novel member of the EXLA family from *C. pallidicaule* seeds. EXLAs belong to the expansin superfamily, a group of extracellular proteins that mediate cell wall loosening and are implicated in plant growth and stress responses. The strategy adopted is particularly relevant given that, to date, no members of the EXLA family have been purified using a classical biochemical approach. This is because EXPLA genes are expressed at trace levels in highly specific, localized tissue types, which means that most functional insights have been inferred from transcriptional analyses and overexpression studies. In addition, the characterization of native proteins allows the study of post-translational modifications.

In light of this, cpEXLA represents a valuable prototype to elucidate the structural and functional mechanism of this enigmatic, nonenzymatic protein family.

On the other hand, the determination of purified cpEXLA primary structure through Edman degradation and cDNA isolation, as well as its further structural characterization combining biochemical and bioinformatic approaches, confirms the distinctive structural features of the EXLA family. Indeed, cpEXLA retains specific conserved residues, five disulfide bonds, and a characteristic two-domain fold, comprising an N-terminal and a C-terminal domain, each with its own conserved amino acid patterns.

## Data Availability

The original contributions presented in this study are included in the article/Supplementary Materials. Further inquiries can be directed to the corresponding author.

## Supplementary Materials

The following supporting information can be downloaded at: https://www.mdpi.com/article/10.3390/ijms27125213/s1. References [73,74,75,76,77] are cited in Supplementary File.
