# Supplementary material for: Purification, Amino Acid Sequence, and Structural Features of a Novel Expansin-like A from the Seeds of Canihua (Chenopodium pallidicaule Aellen)
Source: Int J Mol Sci. 2026 Jun 9;27(12):5213. doi: 10.3390/ijms27125213 (PMC13299222; doi:10.3390/ijms27125213)
Supplement: Supplementary file 1 [file ijms-27-05213-s001.zip › ijms-4359839-supplementary.pdf]

Supplementary materials

Purification, Amino Acid Sequence, and Structural Features of a Novel Expansin-like A from the Seeds of Canihua (*Chenopodium pallidicaule* Aellen)

Sara Ragucci [1,†], Maria Giuseppina Campanile [1,†], Rosario Iglesias [2], Nicola Landi [3], Claudia Carolina Gay [4], Angela Oliver [5], Robina Khan [1], Lucía Citores [2], José Miguel Ferreras [2] and Antimo Di Maro [1,*]

[1] Department of Environmental, Biological and Pharmaceutical Sciences and Technologies (DiSTABiF), University of Campania 'Luigi Vanvitelli', Via Vivaldi 43, 81100-Caserta, Italy; sara.ragucci@unicampania.it (S.R.); mariagiuseppina.campanile@unicampania.it (M.G.C.); robina.khan@unicampania.it (R.K.)

[2] Department of Biochemistry and Molecular Biology and Physiology, Faculty of Sciences, University of Valladolid, 47011 Valladolid, Spain; riglesias@uva.es (R.I.); lucia.citores@uva.es (L.C.); josemiguel.ferreras@uva.es (J.M.F.)

[3] Institute of Crystallography, National Research Council, Via Vivaldi 43, 81100-Caserta, Italy; nicola.landi@unicampania.it

[4] Laboratory of Protein Research, Institute of Basic and Applied Chemistry of Northeast Argentina (UNNE-CONICET), Faculty of Exact and Natural Sciences and Surveying (UNNE), Corrientes 3400, Argentina; claudiacgay@exa.unne.edu.ar

[5] Institute of Biostructures and Bioimaging, National Research Council, Via P. Castellino, 111, 80131 Naples, Italy; angelaoliver@cnr.it

* Correspondence: antimo.dimaro@unicampania.it; Tel.: +39-0823-274409

[†] These authors contributed equally to this work

Table S1. Amino acid compositions of cpEXLA. Residues are expressed as number of residues/mol of protein. Three letter amino acid code has been used.

**Amino acid**	**Residue of cpEXLA by amino acid analysis**	**Residue of cpEXLA by deduced cDNA #**
Ala	23	(23)
Arg	8	(7)
Asx *	22	(20)
Cys	10	(10)
Glx*	17	(15)
Gly	21	(24)
His	2	(2)
Ile	16	(16)
Leu	15	(16)
Lys	19	(21)
Met	5	(4)
Phe	12	(11)
Pro	16	(14)
Ser	18	(16)
Thr	18	(17)
Trp	n.d.	(4)
Tyr	12	(10)
Val	16	(16)
(total)	~(250)	

*, Asx and Glx represent Asp+Asn and Glu+Gln, respectively. #, see main text; n.d.= not determined.

Table S2. Hypothesis of difference among the glycoforms of cpEXLA (see **Fig. S3**) based on Glycomod (https://web.expasy.org/glycomod/) prediction.

Glycoform	Molecular weight (Da)	Difference	Δmass (Da)	Prediction	Note
A *	27569.07	-	-	-	-
B	27731.07	B-A	162.0	Hexose (Hex; 162 Da)	-
C	27949.27	C-B	218.2	N-Acetyl-hexosamine (HexNAc, 203.08 Da)	+16 Da (methionine sulfoxide)
D	28080.77	D-C	131.5	Pentose (Pent; 132 Da)	-
E	28212.77	E-D	132.0	Pent	-
F	28243.37	F-D	162.6	Hex	-
F	28243.37	F-E	~31.0	-	+32 Da (methionine sulfone)
H	28699.57	H-G	324.7	$(Hex)_2$	-

* The molecular weight of glycoform A of cpEXLA is discussed in the main text (paragraph 3.4).

Table S3. Families and orders used for the phylogenetic analysis.

**Orders**	**Families**	**Species**
**Aquifoliales**	Aquifoliaceae	*Ilex paraguariensis* A.St.-Hil.
**Asterales**	Asteraceae	*Arctium lappa* L.
		Centaurea solstitialis L.
		Cynara cardunculus L.
		Erigeron canadensis L.
**Caryophyllales**	Amaranthaceae	*Amaranthus tricolor* L.
		Beta vulgaris L.
		Bienertia sinuspersici Akhani
		Chenopodium quinoa Willd.
		Chenopodium pallidicaule Aellen
		Spinacia oleracea L.
	Cactaceae	*Carnegiea gigantea* (Engelm.) Britton & Rose
	Caryophyllaceae	*Gypsophila vaccaria* (L.) Sm.
		Saponaria officinalis L.
		Silene latifolia Poir.
		Silene pusilla Waldst. & Kit. (= *Heliosperma pusillum* (Waldst. & Kit.) Rchb.)
	Droseraceae	*Dionaea muscipula* J.Ellis
	Nepenthaceae	*Nepenthes gracilis* Korth.
		Nepenthes mirabilis (Lour.) Druce
	Nyctaginaceae	*Mirabilis jalapa* L.
**Celastrales**	Celastraceae	*Tripterygium wilfordii* Hook.f.
**Ericales**	Actinidiaceae	*Actinidia chinensis* Planch.
		Actinidia eriantha Benth.
		Actinidia rufa Franch. & Sav.
	Ebenaceae	*Diospyros lotus* L.
	Ericaceae	*Calluna vulgaris* (L.) Hill
		Rhododendron vialii Delavay & Franch.
		Rhododendron simsii Planch.
		Rhododendron molle (Blume) G.Don
	Lecythidaceae	*Bertholletia excelsa* Bonpl.
**Fabales**	Fabaceae	*Astragalus penduliflorus* Lam. (= *Astragalus alpinus* (L.) E.H.L.Krause)
		Bauhinia variegata L.
		Cajanus cajan (L.) Huth
		Canavalia gladiata (Jacq.) DC.
		Flemingia javanica C.Y.Wu (= *Flemingia macrophylla* Bold.)
		Gastrolobium bilobum R.Br.
		Prosopis cineraria (L.) Druce
		Senna tora (L.) Roxb.
		Spatholobus suberectus Dunn

		Sphenostylis stenocarpa (Hochst. ex A.Rich.) Harms
	Quillajaceae	*Quillaja saponaria* Molina
**Fagales**	Fagaceae	*Fagus crenata* Blume
	Myricaceae	*Myrica rubra* (Lour.) Siebold & Zucc. (= *Morella rubra* Lour.)
**Gentianales**	Rubiaceae	*Cinchona calisaya* Wedd.
**Lamiales**	Bignoniaceae	*Handroanthus impetiginosus* (Mart. ex DC.) Mattos
	Orobanchaceae	*Rehmannia glutinosa* (Gaertn.) Libosch. ex DC.
	Plantaginaceae	*Penstemon davidsonii* Greene
**Malvales**	Dipterocarpaceae	*Rubroshorea leprosula* (Miq.) P.S.Ashton & J.Heck.
		Shorea laevis Ridl.
	Malvaceae	*Corchorus capsularis* L.
		Craigia yunnanensis W.W.Sm. & W.E.Evans
		Cullenia ceylanica (Gardner) Wight ex K.Schum.(= *Durio zibethinus* Moon)
		Pityranthe trichosperma (Merr.) Kubitzki (= *Diplodiscus trichospermus* (Merr.) Y.Tang, M.G.Gilbert & Dorr)
		Gossypium arboreum L. (= *Gossypium anomalum* G.Watt)
		Gossypium aridum (Rose & Standl.) Skovsted
		Gossypium australe F.Muell.
		Gossypium barbadense L.
		Gossypium darwinii G.Watt
		Gossypium harknessii Brandegee
		Gossypium hirsutum L.
		Gossypium harknessii subsp. armourianum (Kearney) Roberty (= *Gossypium armourianum* Kearney)
		Gossypium klotzschianum Andersson
		Gossypium klotzschianum subsp. *davidsonii* (Kellogg) Roberty (= *Gossypium davidsonii* Kellogg)
		Gossypium laxum L.Ll.Phillips
		Gossypium lobatum Gentry
		Gossypium mustelinum Miers ex G.Watt
		Gossypium raimondii Ulbr.
		Gossypium schwendimanii Fryxell & S.D.Koch
		Gossypium stocksii Mast.
		Gossypium sturtianum J.H.Willis (= *Gossypium gossypioides* (R.Br.) C.A.Gardner)
		Gossypium tomentosum Nutt. ex Seem.
		Gossypium trilobum (Sessé & Moc. ex DC.) Skovst.
		Gossypium turneri Fryxell
		Herrania umbratica R.E.Schult.
		Hibiscus cannabinus L.

		Hibiscus sabdariffa L.
		Hibiscus syriacus L.
		Hibiscus trionum L.
		Reevesia pubescens Mast.
		Theobroma cacao L.
**Malpighiales**	Erythroxylaceae	*Erythroxylum novogranatense* (D.Morris) Hieron.
	Euphorbiaceae	Jatropha curcas L.
	Passifloraceae	*Turnera subulata* Sm.
	Salicaceae	*Populus alba* L.
		Populus alba x *Populus* x *berolinensis*
		Populus × *canadensis* Moench
		Populus deltoides W.Bartram ex Marshall
		Populus euphratica Olivier
		Populus nigra L.
		Populus tomentosa Carrière
		Populus trichocarpa Torr. & A.Gray ex Hook.
		Salix lindleyana Wall. ex Andersson (= Salix brachista C.K.Schneid.)
		Salix mesnyi Hance (= *Salix dunnii* C.K.Schneid.)
		Salix suchowensis W.C.Cheng ex G.H.Zhu
		Salix udensis Trautv. & C.A.Mey.
		Salix viminalis L.
**Myrtales**	Lythraceae	*Punica granatum* L.
	Myrtaceae	*Corymbia citriodora* (Hook.) K.D.Hill & L.A.S.Johnson
		Rhodamnia argentea Benth.
		Syzygium grande (Wight) Walp.
		Syzygium oleosum (F.Muell.) B.Hyland
**Oxalidales**	Cephalotaceae	*Cephalotus follicularis* Labill.
**Ranunculales**	Circaeasteraceae	*Kingdonia uniflora* Balf.f. & W.W.Sm.
**Rosales**	Cannabaceae	*Cannabis sativa* L.
		Humulus lupulus L.
		Trema orientale (L.) Blume
	Moraceae	*Morus notabilis* C.K.Schneid.
	Rhamnaceae	*Rhamnella rubrinervis* (H.Lév.) Rehder
**Sapindales**	Anacardiaceae	*Mangifera indica* L.
		Pistacia atlantica Desf.
		Pistacia vera L.
**Saxifragales**	Crassulaceae	*Rhodiola kirilowii* (Regel) Maxim.
**Solanales**	Convolvulaceae	*Cuscuta australis* R.Br.
		Cuscuta campestris Yunck.
		Ipomoea batatas (L.) Lam.
		Ipomoea nil (L.) Roth
		Ipomoea triloba L.
	Solanaceae	Nicotiana attenuata Torr. ex S.Watson

		Nicotiana sylvestris Speg.
		Nicotiana tabacum L.
		Nicotiana tomentosiformis Goodsp.

Figure S1.

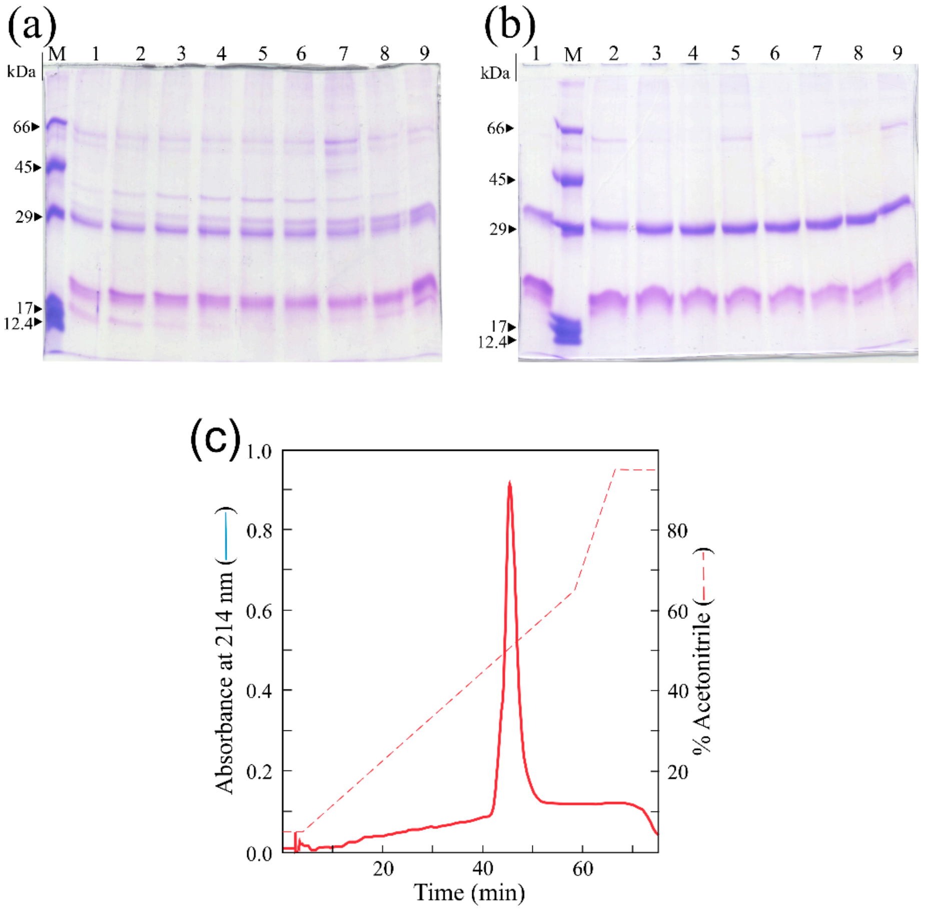

Fig. S1. (**a**) and (**b**) SDS-PAGE analysis of fractions (3.0 μg) 136-144 (corresponding to lane 1-9) and 206-214 (corresponding to lane 1-9) from peak CM-1 and CM-2, respectively, obtained after cation exchange chromatography using CM-Sepharose resin. M, molecular weight markers. SDS-PAGE in the presence of β-mercaptoethanol was carried out in 12% polyacrylamide separating gel and then stained with Coomassie brilliant blue. (**c**) RP-HPLC chromatographic profile of cpEXLA (~100 μg) separated by C-4 analytical column.

Figure S2.

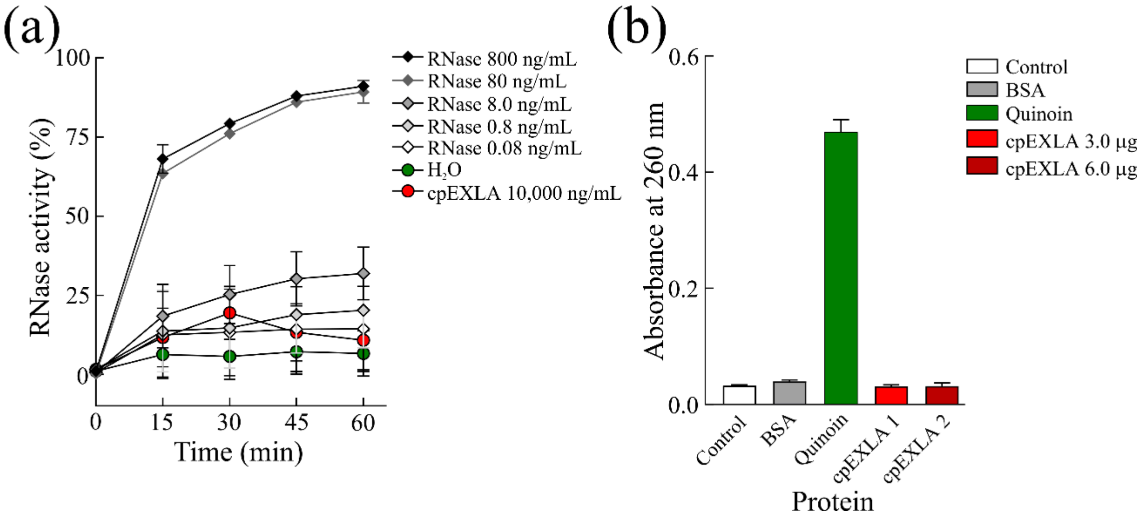

Fig. S2. (**a**) Ribonuclease activity of cpEXLA compared with ribonuclease A from bovine pancreas. Yeast RNA was incubated with the protein concentrations indicated in the Figure as described in Materials and Methods, and RNA degradation was estimated by monitoring the decrease in absorbance at 688 nm. (**b**) Polynucleotide:adenosine glycosylase activity of BSA (negative control), quinoin (positive control) and cpEXLA. Proteins (concentrations indicated in the Figure) were incubated with salmon sperm DNA, as reported in the Materials and Methods section. Control, salmon sperm DNA incubated with water. The mean results ± SD from three independent experiments performed in triplicate are shown.

Figure S3.

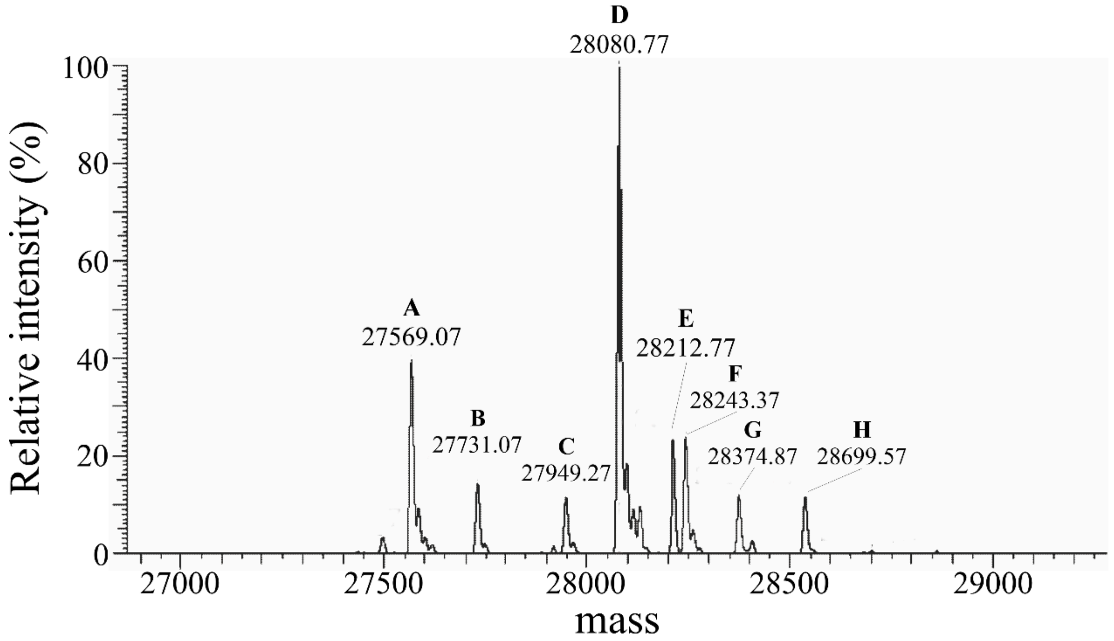

Fig. S3. Deconvoluted mass spectrum of RP-HPLC purified cpEXLA acquired by LTQ Linear Ion Trap mass spectrometer.

Figure S4.

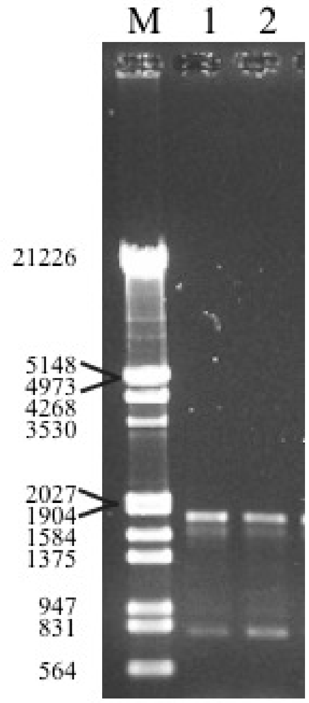

Fig. S4. PCR amplification of the cpEXLA cDNA. Amplicons with (top) and without (bottom) introns are shown (1-2, duplicates). The numbers indicate the size of the markers (M, Lambda DNAEcoR I/Hind III double digest) in base pairs.

Figure S5.

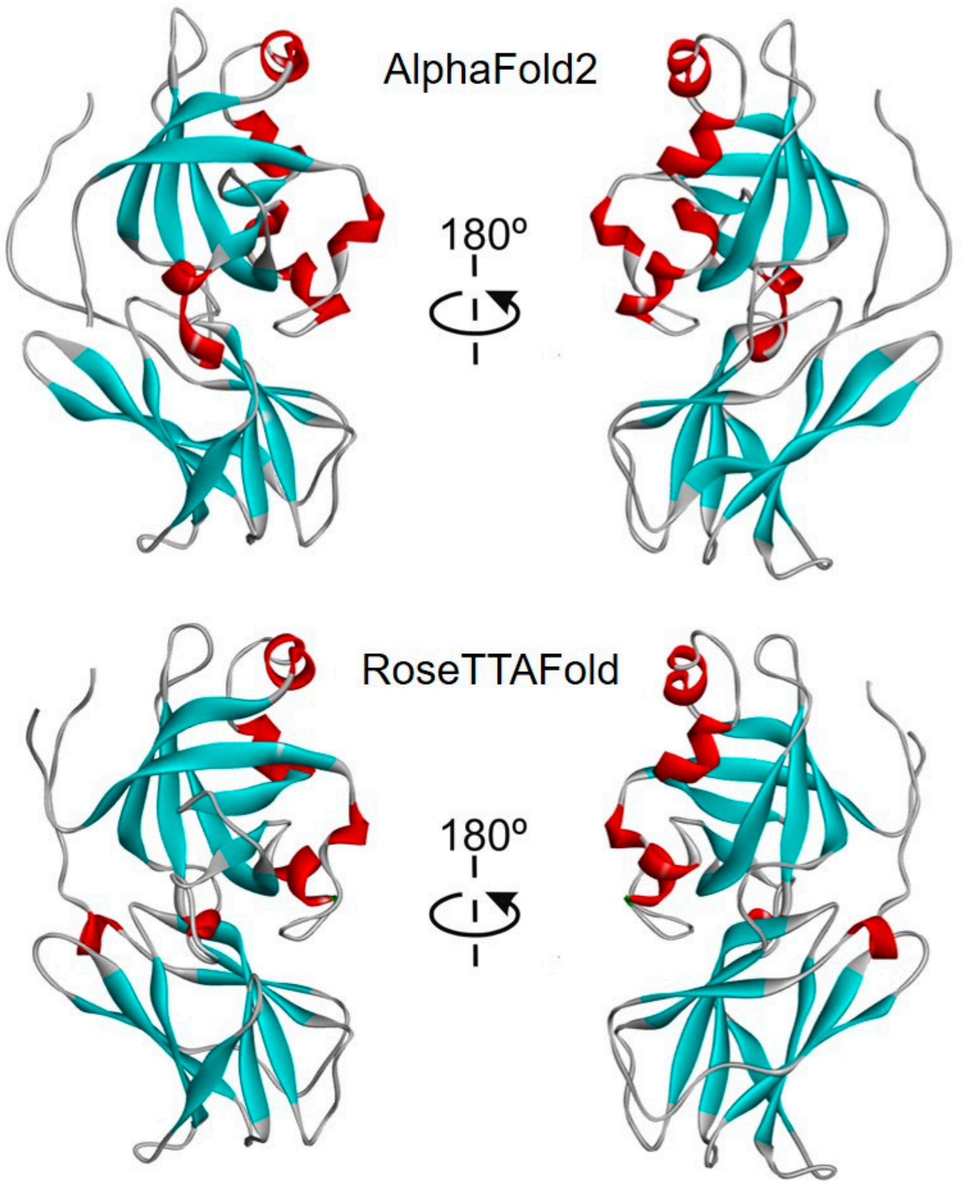

Fig. S5. Comparison of the predictions of the structure of cpEXLA made by AlphaFold2 (upper panel) and RoseTTAFold (lower panel).

Figure S6.

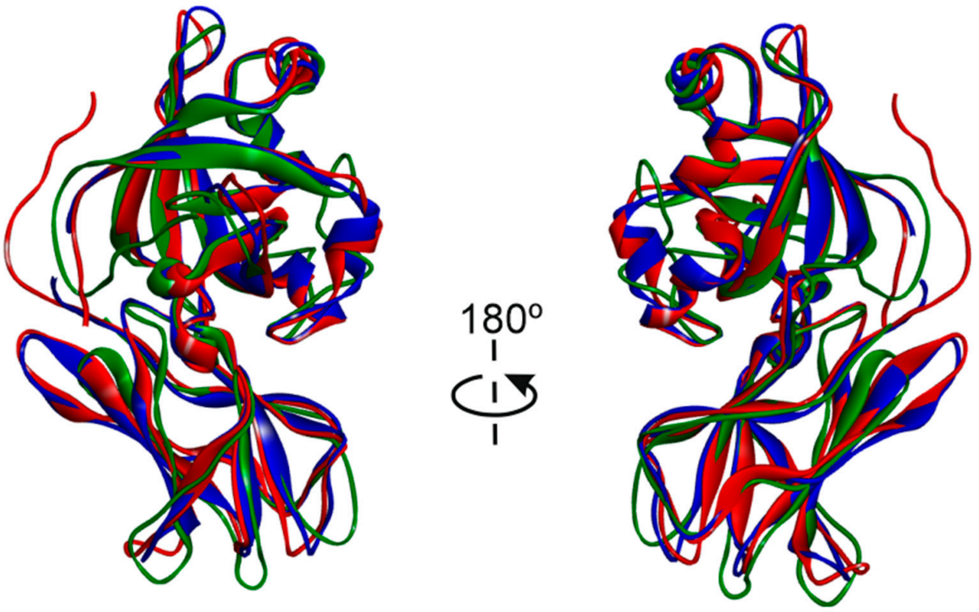

Fig. S6. Comparison of the structures of cpEXLA (red), alpha-like expansin GhEXLA1 (blue), and beta-expansin EXPB1 (Zea m 1) (green). The alignment of the structures was performed using TM-align, as indicated in the Materials and Methods section.

Figure S7.

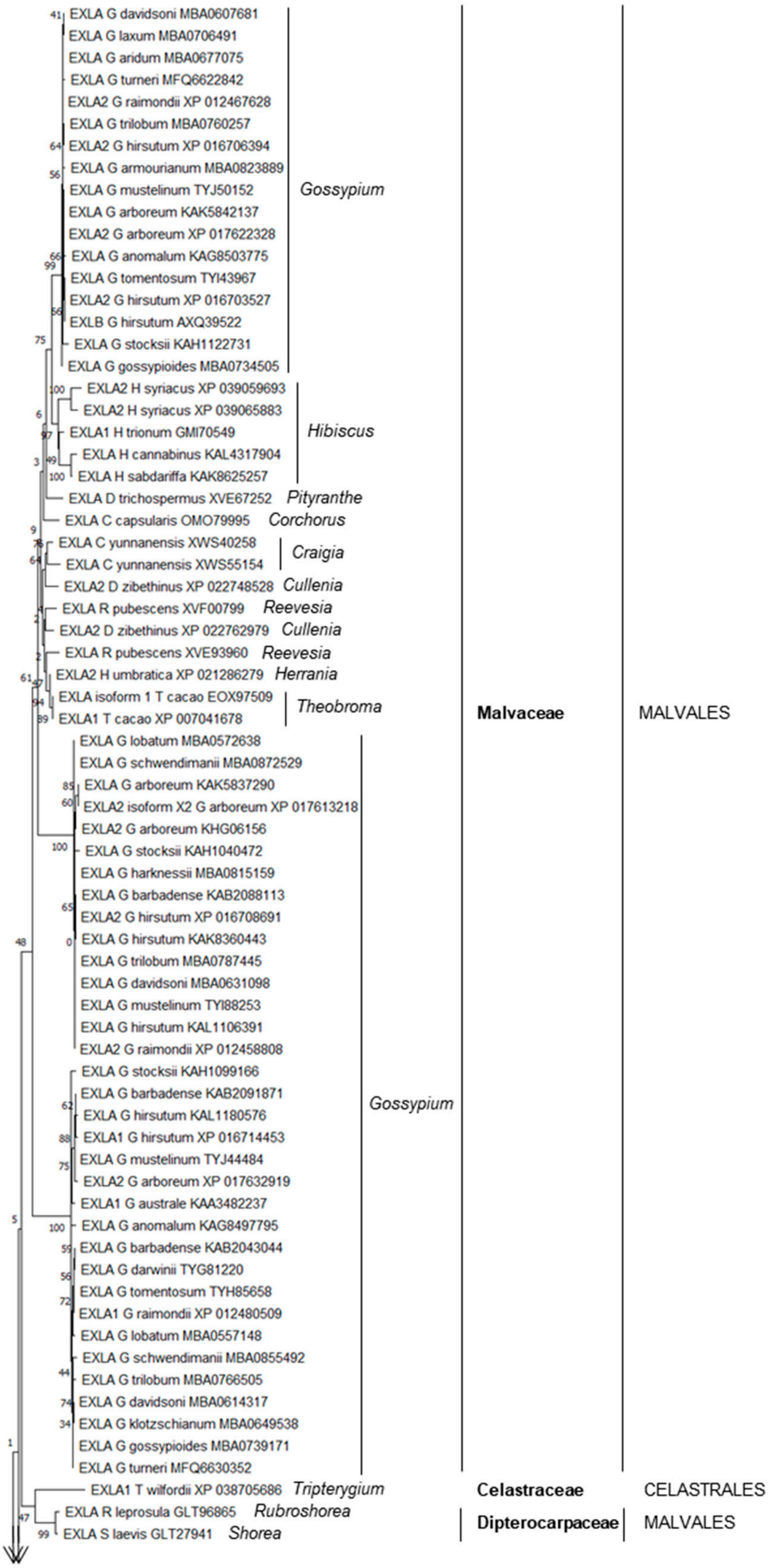
EXLA G davidsoni MBA0607681
EXLA G laxum MBA0706491
EXLA G aridum MBA0677075
EXLA G turneri MFQ6622842
EXLA2 G raimondii XP 012467628
EXLA G trilobum MBA0760257
EXLA2 G hirsutum XP 016706394
EXLA G armourianum MBA0823889
EXLA G mustelinum TYJ50152
EXLA G arboreum KAK5842137
EXLA2 G arboreum XP 017622328
EXLA G anomalum KAG8503775
EXLA G tomentosum TYI43967
EXLA2 G hirsutum XP 016703527
EXLB G hirsutum AXQ39522
EXLA G stocksii KAH1122731
EXLA G gossypioides MBA0734505
Gossypium
EXLA2 H syriacus XP 039059693
EXLA2 H syriacus XP 039065883
EXLA1 H trionum GMI70549
EXLA H cannabinus KAL4317904
EXLA H sabdariffa KAK8625257
Hibiscus
EXLA D trichospermus XVE67252 Pityranthe
EXLA C capsularis OMO79995 Corchorus
EXLA C yunnanensis XWS40258
EXLA C yunnanensis XWS55154
Craigia
EXLA2 D zibethinus XP 022748528 Cullenia
EXLA R pubescens XVF00799 Reevesia
EXLA2 D zibethinus XP 022762979 Cullenia
EXLA R pubescens XVE93960 Reevesia
EXLA2 H umbratica XP 021286279 Herrania
EXLA isoform 1 T cacao EOX97509
EXLA1 T cacao XP 007041678
Theobroma
EXLA G lobatum MBA0572638
EXLA G schwendimanii MBA0872529
EXLA G arboreum KAK5837290
EXLA2 isoform X2 G arboreum XP 017613218
EXLA2 G arboreum KHG06156
EXLA G stocksii KAH1040472
EXLA G harknessii MBA0815159
EXLA G barbadense KAB2088113
EXLA2 G hirsutum XP 016708691
EXLA G hirsutum KAK8360443
EXLA G trilobum MBA0787445
EXLA G davidsoni MBA0631098
EXLA G mustelinum TYI88253
EXLA G hirsutum KAL1106391
EXLA2 G raimondii XP 012458808
EXLA G stocksii KAH1099166
EXLA G barbadense KAB2091871
EXLA G hirsutum KAL1180576
EXLA1 G hirsutum XP 016714453
EXLA G mustelinum TYJ44484
EXLA2 G arboreum XP 017632919
EXLA1 G australe KAA3482237
EXLA G anomalum KAG8497795
EXLA G barbadense KAB2043044
EXLA G darwinii TYG81220
EXLA G tomentosum TYH85658
EXLA1 G raimondii XP 012480509
EXLA G lobatum MBA0557148
EXLA G schwendimanii MBA0855492
EXLA G trilobum MBA0766505
EXLA G davidsoni MBA0614317
EXLA G klotzschianum MBA0649538
EXLA G gossypioides MBA0739171
EXLA G turneri MFQ6630352
Gossypium
Malvaceae
MALVALES
EXLA1 T wilfordii XP 038705686 Tripterygium
Celastraceae
CELASTRALES
EXLA R leprosula GLT96865 Rubroshorea
EXLA S laevis GLT27941 Shorea
Dipterocarpaceae
MALVALES

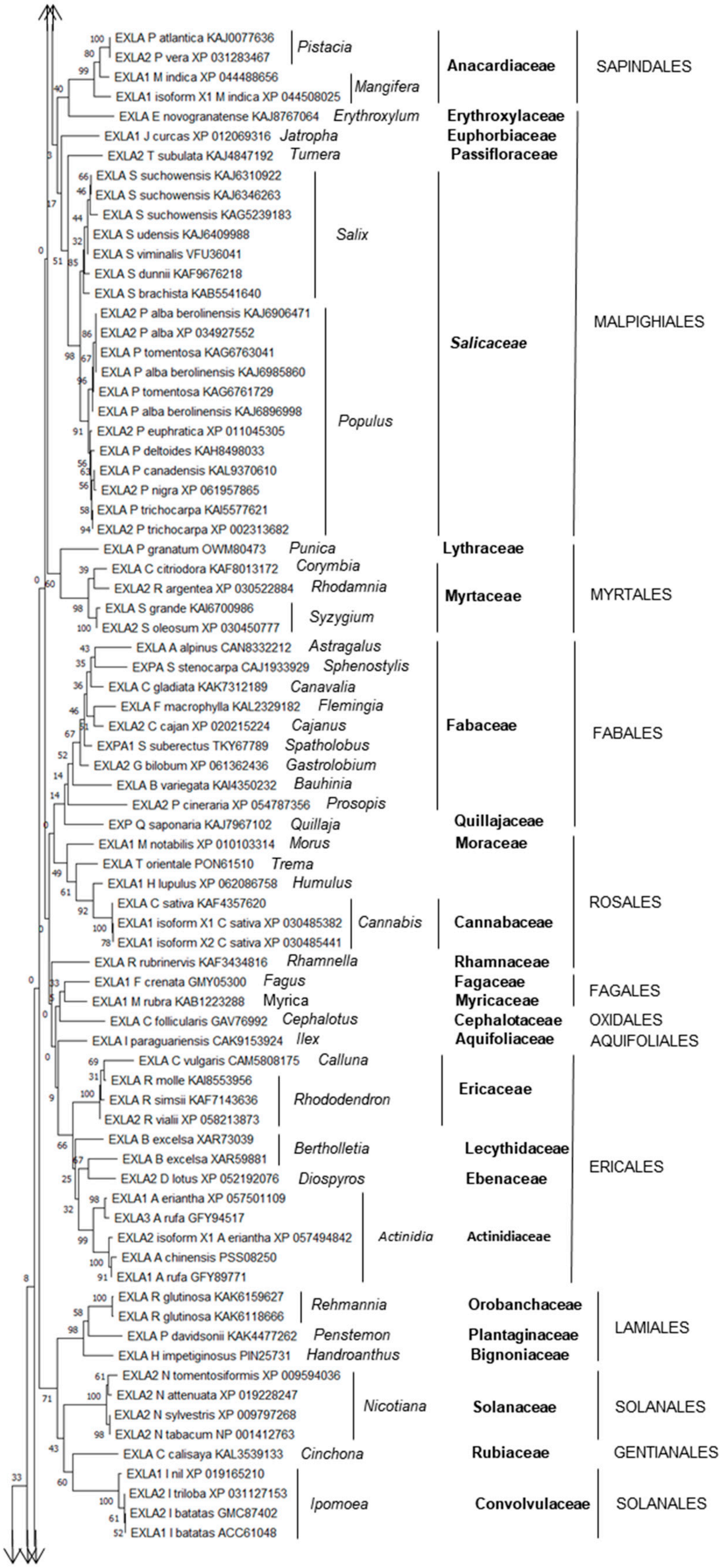
EXLA P atlantica KAJ0077636
EXLA2 P vera XP 031283467
Pistacia
EXLA1 M indica XP 044488656
EXLA1 isoform X1 M indica XP 044508025
Mangifera
Anacardiaceae
SAPINDALES
EXLA E novogranatense KAJ8767064
Erythroxylum
Erythroxylaceae
EXLA1 J curcas XP 012069316
Jatropha
Euphorbiaceae
EXLA2 T subulata KAJ4847192
Turnera
Passifloraceae
EXLA S suchowensis KAJ6310922
EXLA S suchowensis KAJ6346263
EXLA S suchowensis KAG5239183
EXLA S udensis KAJ6409988
EXLA S viminalis VFU36041
EXLA S dunnii KAF9676218
EXLA S brachista KAB5541640
Salix
EXLA2 P alba berolinensis KAJ6906471
EXLA2 P alba XP 034927552
EXLA P tomentosa KAG6763041
EXLA P alba berolinensis KAJ6985860
EXLA P tomentosa KAG6761729
EXLA P alba berolinensis KAJ6896998
EXLA2 P euphratica XP 011045305
EXLA P deltoides KAH8498033
EXLA P canadensis KAL9370610
EXLA2 P nigra XP 061957865
EXLA P trichocarpa KAI5577621
EXLA2 P trichocarpa XP 002313682
Populus
Salicaceae
MALPIGHIALES
EXLA P granatum OWM80473
Punica
Lythraceae
EXLA C citriodora KAF8013172
Corymbia
EXLA2 R argentea XP 030522884
Rhodamnia
EXLA S grande KAI6700986
EXLA2 S oleosum XP 030450777
Syzygium
Myrtaceae
MYRTALES
EXLA A alpinus CAN8332212
Astragalus
EXPA S stenocarpa CAJ1933929
Sphenostylis
EXLA C gladiata KAK7312189
Canavalia
EXLA F macrophylla KAL2329182
Flemingia
EXLA2 C cajan XP 020215224
Cajanus
EXPA1 S suberectus TKY67789
Spatholobus
EXLA2 G bilobum XP 061362436
Gastrolobium
EXLA B variegata KAI4350232
Bauhinia
EXLA2 P cineraria XP 054787356
Prosopis
Fabaceae
FABALES
EXP Q saponaria KAJ7967102
Quillaja
Quillajaceae
EXLA1 M notabilis XP 010103314
Morus
Moraceae
EXLA T orientale PON61510
Trema
EXLA1 H lupulus XP 062086758
Humulus
EXLA C sativa KAF4357620
EXLA1 isoform X1 C sativa XP 030485382
EXLA1 isoform X2 C sativa XP 030485441
Cannabis
Cannabaceae
ROSALES
EXLA R rubrinervis KAF3434816
Rhamnella
Rhamnaceae
EXLA1 F crenata GMY05300
Fagus
Fagaceae
EXLA1 M rubra KAB1223288
Myrica
Myricaceae
FAGALES
EXLA C follicularis GAV76992
Cephalotus
Cephalotaceae
OXIDALES
EXLA I paraguariensis CAK9153924
Ilex
Aquifoliaceae
AQUIFOLIALES
EXLA C vulgaris CAM5808175
Calluna
EXLA R molle KAI8553956
EXLA R simsii KAF7143636
EXLA2 R vialii XP 058213873
Rhododendron
Ericaceae
EXLA B excelsa XAR73039
EXLA B excelsa XAR59881
Bertholletia
Lecythidaceae
EXLA2 D lotus XP 052192076
Diospyros
Ebenaceae
EXLA1 A eriantha XP 057501109
EXLA3 A rufa GFY94517
EXLA2 isoform X1 A eriantha XP 057494842
EXLA A chinensis PSS08250
EXLA1 A rufa GFY89771
Actinidia
Actinidiaceae
ERICALES
EXLA R glutinosa KAK6159627
EXLA R glutinosa KAK6118666
Rehmannia
Orobanchaceae
EXLA P davidsonii KAK4477262
Penstemon
Plantaginaceae
EXLA H impetiginosus PIN25731
Handroanthus
Bignoniaceae
LAMIALES
EXLA2 N tomentosiformis XP 009594036
EXLA2 N attenuata XP 019228247
EXLA2 N sylvestris XP 009797268
EXLA2 N tabacum NP 001412763
Nicotiana
Solanaceae
SOLANALES
EXLA C calisaya KAL3539133
Cinchona
Rubiaceae
GENTIANALES
EXLA1 I nil XP 019165210
EXLA2 I triloba XP 031127153
EXLA2 I batatas GMC87402
EXLA1 I batatas ACC61048
Ipomoea
Convolvulaceae
SOLANALES

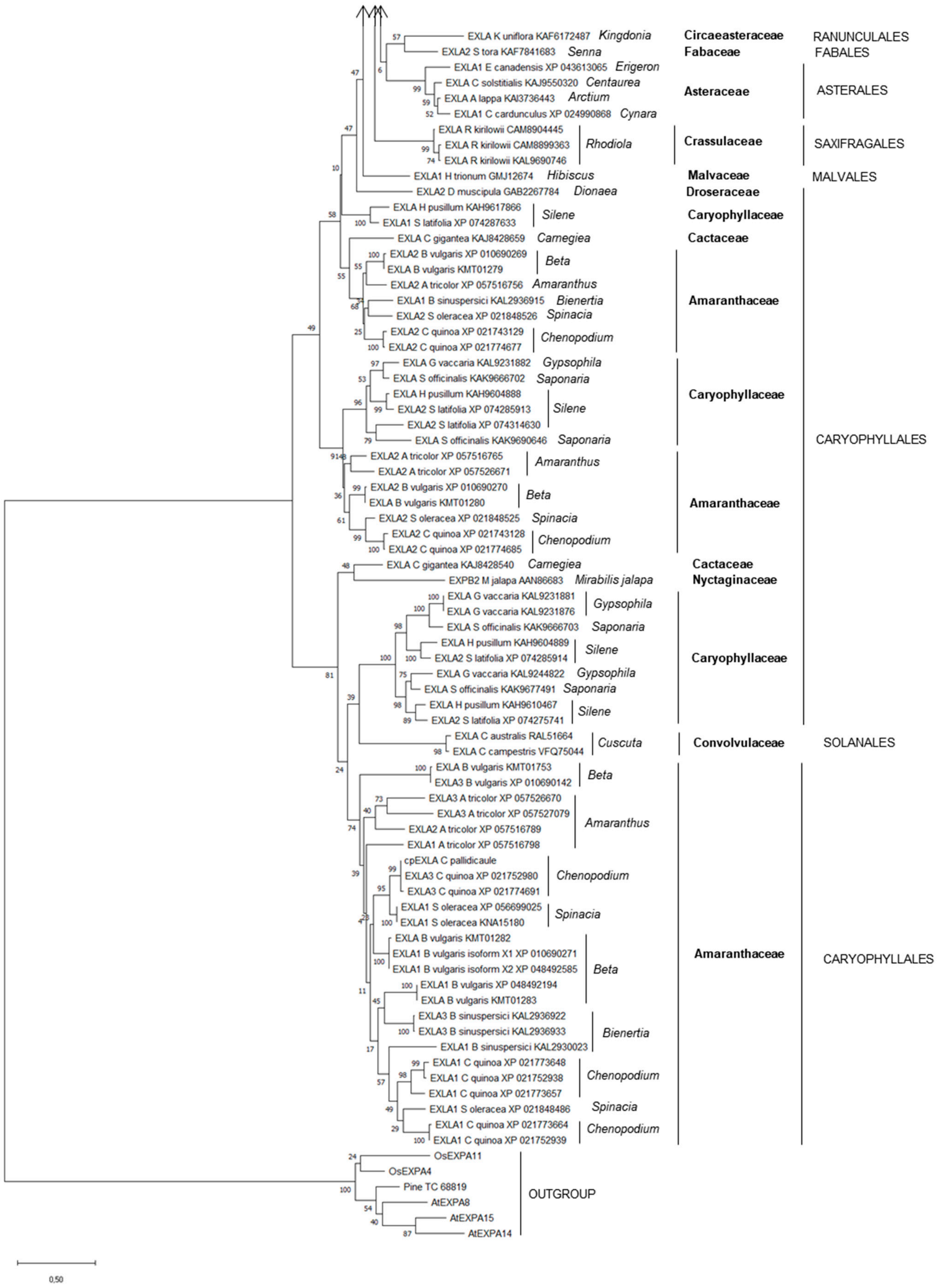

EXLA K uniflora KAF6172487 Kingdonia
EXLA2 S tora KAF7841683 Senna
EXLA1 E canadensis XP 043613065 Erigeron
EXLA C solstitialis KAJ9550320 Centaurea
EXLA A lappa KAI3736443 Arctium
EXLA1 C cardunculus XP 024990868 Cynara
EXLA R kirilowii CAM8904445
EXLA R kirilowii CAM8899363 Rhodiola
EXLA R kirilowii KAL9690746
EXLA1 H trionum GMJ12674 Hibiscus
EXLA2 D muscipula GAB2267784 Dionaea
EXLA H pusillum KAH9617866 Silene
EXLA1 S latifolia XP 074287633
EXLA C gigantea KAJ8428659 Carnegiea
EXLA2 B vulgaris XP 010690269 Beta
EXLA B vulgaris KMT01279
EXLA2 A tricolor XP 057516756 Amaranthus
EXLA1 B sinuspersici KAL2936915 Bienertia
EXLA2 S oleracea XP 021848526 Spinacia
EXLA2 C quinoa XP 021743129 Chenopodium
EXLA2 C quinoa XP 021774677
EXLA G vaccaria KAL9231882 Gypsophila
EXLA S officinalis KAK9666702 Saponaria
EXLA H pusillum KAH9604888 Silene
EXLA2 S latifolia XP 074285913
EXLA2 S latifolia XP 074314630
EXLA S officinalis KAK9690646 Saponaria
EXLA2 A tricolor XP 057516765 Amaranthus
EXLA2 A tricolor XP 057526671
EXLA2 B vulgaris XP 010690270 Beta
EXLA B vulgaris KMT01280
EXLA2 S oleracea XP 021848525 Spinacia
EXLA2 C quinoa XP 021743128 Chenopodium
EXLA2 C quinoa XP 021774685
EXLA C gigantea KAJ8428540 Carnegiea
EXPB2 M jalapa AAN86683 Mirabilis jalapa
EXLA G vaccaria KAL9231881 Gypsophila
EXLA G vaccaria KAL9231876
EXLA S officinalis KAK9666703 Saponaria
EXLA H pusillum KAH9604889 Silene
EXLA2 S latifolia XP 074285914
EXLA G vaccaria KAL9244822 Gypsophila
EXLA S officinalis KAK9677491 Saponaria
EXLA H pusillum KAH9610467 Silene
EXLA2 S latifolia XP 074275741
EXLA C australis RAL51664 Cuscuta
EXLA C campestris VFQ75044
EXLA B vulgaris KMT01753 Beta
EXLA3 B vulgaris XP 010690142
EXLA3 A tricolor XP 057526670
EXLA3 A tricolor XP 057527079 Amaranthus
EXLA2 A tricolor XP 057516789
EXLA1 A tricolor XP 057516798
cpEXLA C pallidicaule
EXLA3 C quinoa XP 021752980 Chenopodium
EXLA3 C quinoa XP 021774691
EXLA1 S oleracea XP 056699025 Spinacia
EXLA1 S oleracea KNA15180
EXLA B vulgaris KMT01282
EXLA1 B vulgaris isoform X1 XP 010690271
EXLA1 B vulgaris isoform X2 XP 048492585 Beta
EXLA1 B vulgaris XP 048492194
EXLA B vulgaris KMT01283
EXLA3 B sinuspersici KAL2936922
EXLA3 B sinuspersici KAL2936933 Bienertia
EXLA1 B sinuspersici KAL2930023
EXLA1 C quinoa XP 021773648
EXLA1 C quinoa XP 021752938 Chenopodium
EXLA1 C quinoa XP 021773657
EXLA1 S oleracea XP 021848486 Spinacia
EXLA1 C quinoa XP 021773664 Chenopodium
EXLA1 C quinoa XP 021752939
OsEXPA11
OsEXPA4
Pine TC 68819
AtEXPA8
AtEXPA15
AtEXPA14
OUTGROUP
Circaeasteraceae
Fabaceae
Asteraceae
Crassulaceae
Malvaceae
Droseraceae
Caryophyllaceae
Cactaceae
Amaranthaceae
Caryophyllaceae
Amaranthaceae
Cactaceae
Nyctaginaceae
Caryophyllaceae
Convolvulaceae
Amaranthaceae
RANUNCULALES
FABALES
ASTERALES
SAXIFRAGALES
MALVALES
CARYOPHYLLALES
SOLANALES
CARYOPHYLLALES
0,50

Fig. S7. Phylogenetic analysis using the maximum likelihood method of the precursors of expansin-like protein A (EXLA) in angiosperms. The phylogeny was deduced using the maximum likelihood method and the Jones-Taylor-Thornton amino acid substitution model [73]. The tree with the highest logarithmic likelihood (-25,064.58) is shown. The percentage of replicated trees in which the associated taxa were grouped (500 replicates) is shown next to the branches [74]. The initial trees for the heuristic search were obtained automatically by applying the Neighbor-Joining [75] and BioNJ [76] algorithms to a matrix of pairwise distances estimated using the maximum composite likelihood (MCL) approach [77] and then selecting the topology with the highest log-likelihood value. Differences in the rate of evolution between sites were modeled using a discrete gamma distribution in 5 categories (+G, parameter = 1.0292). The analytical procedure encompassed 225 amino acid sequences in 315 positions in the final dataset. Evolutionary analyses were performed in MEGA11 [69]. The sequence name, species, genus, family, order, and accession number are indicated.

Figure S8.

1. >OsEXPA11 [Oryza sativa Japonica Group]
MELLRLLAVAAVAAMAAEVAAGGDSGWSSGSATFYGGSDASGTMGGACGYGNLYSAGYGTSTAALSTALFNNGQSCG
ACFEVRCGGGGSCLAGTVAVTATNLCPPNYALAGDAGGWCNPPRPHFDMAEPAFTRIAQARAGVVPVQYRRVACAKQ
GGIRFTITGHSYFNLVLVTNVGGAGDVTAVSVKGSRSGWQAMSHNWGANWQNGANLDGQPLSFRVTASDGRTVTSDN
VAPSGWSFGQTFSGGQF

2. >AtEXPA15 [Arabidopsis thaliana]
MFMGKMGLLGIALFCFAAMVCSVHGYDAGWVNAHATFYGGSDASGTMGGACGYGNLYSQGYGTNTAALSTALFNNGL
SCGACFEIKCQSDGAWCLPGAIIVTATNFCPPNNALPNNAGGWCNPPLHHFDLSQPVFQRIAQYKAGVVPVSYRRVP
CMRRGGIRFTINGHSYFNLVLVTNVGGAGDVHSVAVKGSRTRWQQMSRNWGQNWQSNNLLNGQALSFKVTASDGRTV
VSNNIAPASWSFGQTFTGRQFR

3. >AtEXPA14 [Arabidopsis thaliana]
MEFFGKMIISLSLMMMIMWKSVDGYSSGWVNARATFYGGADASGTMGGACGYGNLYSQGYGTNTAALSTALFNGGQS
CGACFQIKCVDDPKWCIGGTITVTGTNFCPPNFAQANNAGGWCNPPQHHFDLAQPIFLRIAQYKAGVVPVQYRRVAC
RRKGGIRFTINGHSYFNLVLITNVAGAGDVISVSIKGTNTRWQSMSRNWGQNWQSNAKLDGQALSFKVTTSDGRTVI
SNNATPRNWSFGQTYTGKQFRAQR

4. >OsEXPA4 [Oryza sativa Japonica Group]
MAIAGVLFLLFLARQASAAGYGGWQSAHATFYGGGDASGTMGGACGYGNLYSQGYGTNTAALSTALFNDGAACGSCY
ELRCDNAGSSCLPGSITVTATNFCPPNYGLPSDDGGWCNPPRPHFDMAEPAFLHIAQYRAGIVPVSFRRVPCVKKGG
VRFTVNGHSYFNLVLVTNVAGAGDVRSVSIKGSRTGWQPMSRNWGQNWQSNAFLDGQSLSFQVTASDGRTVTSNNVA
HPGWQFGQTFEGGQF

5. >Pine_TC_68819 [Pinus taeda]
MRSMELVKSIALASLLTFIWLLTGAHGYGGWESAHATFYGGSDASGTMGGACGYGNLYSQGYGTNTAALSTALFNDG
LSCGACYEMRCNDDPQWCLPGTVTVTATNFCPPNNALPNDNGGWCNPPLQHFDMAEPAFLKIAKYRGGIVPILYTRV
PCLRKGGIRFTVNGHSYFNLVLITNVGGAGDVHAVSIKGSRSGWQPMSRNWGQNWQSNSYLNGQSLSFQVTTSDGRT
VVSNNVAPSNWQFGQTFEGSQV

6. >AtEXPA8 [Arabidopsis thaliana]
MYTPSYLKYSIISIISVLFLQGTHGDDGGWQGGHATFYGGEDASGTMGGACGYGNLYGQGYGTNTAALSTALFNNGL
TCGACYEMKCNDDPRWCLGSTITVTATNFCPPNPGLSNDNGGWCNPPLQHFDLAEPAFLQIAQYRAGIVPVSFRRVP
CMKKGGIRFTINGHSYFNLVLISNVGGAGDVHAVSIKGSKTQSWQAMSRNWGQNWQSNSYMNDQSLSFQVTTSDGRT
LVSNDVAPSNWQFGQTYQGGQF

Fig. S8. Outgroup with 13 EXPA precursors for the phylogenetic analysis

References supplementary material

73. Jones, D. T.; Taylor, W. R.; Thornton, J. M., The rapid generation of mutation data matrices from protein sequences. *Comput Appl Biosci* **1992,** 8, (3), 275-82.
74. Felsenstein, J., Confidence Limits on Phylogenies: An Approach Using the Bootstrap. *Evolution* **1985,** 39, (4), 783-791.
75. Saitou, N.; Nei, M., The neighbor-joining method: a new method for reconstructing phylogenetic trees. *Mol Biol Evol* **1987,** 4, (4), 406-25.
76. Gascuel, O., BIONJ: an improved version of the NJ algorithm based on a simple model of sequence data. *Mol Biol Evol* **1997,** 14, (7), 685-95.
77. Tamura, K.; Nei, M.; Kumar, S., Prospects for inferring very large phylogenies by using the neighbor-joining method. *Proc Natl Acad Sci USA* **2004,** 101, (30), 11030-5.
